# Discovery of a signaling feedback circuit that defines interferon responses in myeloproliferative neoplasms

**DOI:** 10.1038/s41467-022-29381-7

**Published:** 2022-04-01

**Authors:** Diana Saleiro, Jeremy Q. Wen, Ewa M. Kosciuczuk, Frank Eckerdt, Elspeth M. Beauchamp, Chidera V. Oku, Gavin T. Blyth, Mariafausta Fischietti, Liliana Ilut, Marco Colamonici, William Palivos, Paula A. Atsaves, Dean Tan, Masha Kocherginsky, Rona Singer Weinberg, Eleanor N. Fish, John D. Crispino, Ronald Hoffman, Leonidas C. Platanias

**Affiliations:** 1 Robert H. Lurie Comprehensive Cancer Center of Northwestern University, Chicago, IL USA; 2 Division of Hematology-Oncology, Department of Medicine, Feinberg School of Medicine, Northwestern University, Chicago, IL USA; 3 Department of Medicine, Jesse Brown Veterans Affairs Medical Center, Chicago, IL USA; 4 Department of Neurological Surgery, Feinberg School of Medicine, Northwestern University, Chicago, IL USA; 5 Division of Biostatistics, Department of Preventive Medicine, Feinberg School of Medicine, Northwestern University, Chicago, IL USA; 6 New York Blood Center, New York, NY USA; 7 Myeloproliferative Neoplasms Research Consortium, New York, NY USA; 8 Toronto General Hospital Research Institute, University Health Network & Department of Immunology, University of Toronto, Toronto, ON Canada; 9 Tisch Cancer Institute, Icahn School of Medicine at Mount Sinai, New York, NY USA

**Keywords:** Extracellular signalling molecules, Myeloproliferative disease, Signal transduction, Interferons

## Abstract

Interferons (IFNs) are key initiators and effectors of the immune response against malignant cells and also directly inhibit tumor growth. IFNα is highly effective in the treatment of myeloproliferative neoplasms (MPNs), but the mechanisms of action are unclear and it remains unknown why some patients respond to IFNα and others do not. Here, we identify and characterize a pathway involving PKCδ-dependent phosphorylation of ULK1 on serine residues 341 and 495, required for subsequent activation of p38 MAPK. We show that this pathway is essential for IFN-suppressive effects on primary malignant erythroid precursors from MPN patients, and that increased levels of ULK1 and p38 MAPK correlate with clinical response to IFNα therapy in these patients. We also demonstrate that IFNα treatment induces cleavage/activation of the ULK1-interacting ROCK1/2 proteins in vitro and in vivo, triggering a negative feedback loop that suppresses IFN responses. Overexpression of ROCK1/2 is seen in MPN patients and their genetic or pharmacological inhibition enhances IFN-anti-neoplastic responses in malignant erythroid precursors from MPN patients. These findings suggest the clinical potential of pharmacological inhibition of ROCK1/2 in combination with IFN-therapy for the treatment of MPNs.

## Abstract (web summary)

Interferon alpha (IFNalpha) therapy is showing promising results to treat myeloproliferative neoplasms (MPNs). Here, the authors show that IFNalpha response requires ULK1 phosphorylation to induce p38-MAPK signalling but it is counteracted by ROCK1-2 activation suggesting combination therapy of IFNalpha-ROCK1-2 inhibition may improve MPNs treatment.

## Introduction

Type I interferons (IFNs) are critical frontline effectors of the immune defense against pathogens and cancer^1,2^. Additionally, IFNs act directly on tumor cells blocking their growth, survival, migration, and other pro-tumorigenic events^3^. Type I IFNs exert their effects by binding to the Type I IFN receptor (IFNAR) and subsequently activating multiple signaling pathways that lead to the expression of IFN-stimulated genes (ISGs), whose protein products drive several distinct biological functions^4–6^. Extensive clinical studies using different formulations of IFN-alpha (IFNα) have demonstrated anti-tumor activity against several malignancies. IFNα treatment is especially effective in Philadelphia chromosome negative myeloproliferative neoplasms (MPNs), and has been approved by the U.S. Food and Drug Administration for the treatment of hairy cell leukemia, AIDS-related Kaposi’s sarcoma, and chronic myelogenous leukemia (CML)^3,7^.

MPNs are clonal hematopoietic stem cell (HSC) disorders characterized by an increased risk of bleeding, thrombosis, bone marrow fibrosis and, in some cases, transformation to acute myeloid leukemia (AML)^8,9^. There are three types of MPNs: polycythemia vera (PV), essential thrombocythemia (ET), and primary myelofibrosis (PMF). In 95% of MPN patients, somatic mutations in one of three genes: *JAK2*, *CALR*, or *MPL* occur in a single HSC, giving rise to malignant stem cells with constitutively active MPL-JAK-STAT signaling^8^. *JAK2*^V617F^ is the most common mutation and occurs in ~95% of PV patients and 50–60% of ET and PMF patients^10–13^. JAK2 inhibitors are approved for the treatment of MPNs, however, overall they have a modest effect and rarely achieve complete molecular or pathologic remission^14^. In contrast, in recent clinical studies, pegylated IFNα (PEG-IFN) was shown to induce durable hematological and molecular responses in MPN patients, and in some cases complete remission^7,15–19^. The mechanism(s) of action of IFN in MPNs are unclear and the reason for molecular relapses, which have been reported in some patients^20^, are unknown.

We have previously demonstrated that Unc-51-like kinase 1 (ULK1) is required for IFN-induced anti-clonogenic effects against malignant erythroid progenitor cells from MPN patients and that ULK1 is required for IFN-mediated activation of p38 MAPK and expression of specific ISGs^21^. In the present study, we identify protein kinase C-delta (PKCδ) as an upstream regulator of the ULK1-p38 MAPK cascade. We also identify ROCK1/2 as interactor proteins of ULK1 and negative feedback regulators of IFNα-induced anti-neoplastic effects in primary MPN cells. Additionally, we provide evidence that increased expression of elements of the pathway correlate with clinical response to IFNα therapy in MPN patients, underscoring the importance of this signaling circuit in the IFN-system.

## Results

### Type I IFN treatment activates a PKCδ-ULK1-p38 MAPK signaling cascade

**Fig. 1:**
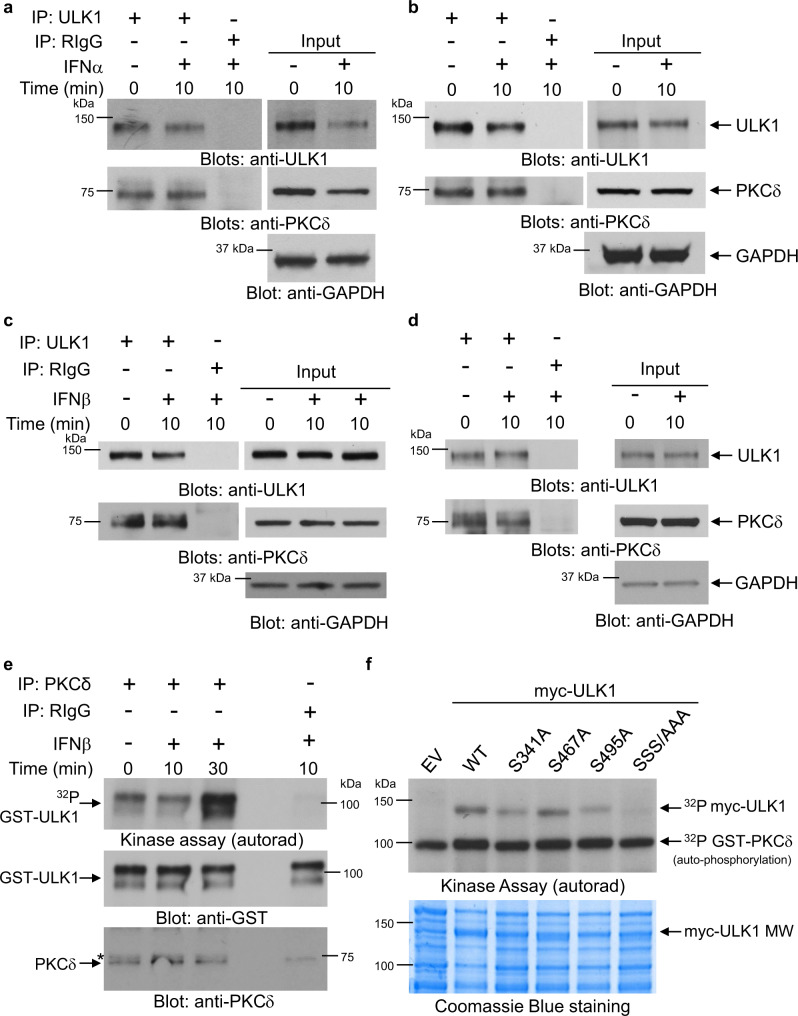
PKCδ binds to and directly phosphorylates ULK1 on serine residues 341 and 495. **a**–**d** Immunoblots for the indicated proteins in total cell lysates (input) or immunoprecipitates (IP) with anti-ULK1 or RIgG (negative control) antibodies from (**a**) HEL, (**b**) SET-2, (**c**) KT-1, and (**d**) U937 cells, left untreated or treated with IFNα or IFNβ (10^4^ IU/mL) for 10 min, as indicated. Blots are representative of two independent experiments for each cell line. RIgG, normal Rabbit Immunoglobulin G. IP samples and corresponding total cell lysates (input) were resolved and blotted from the same gel as shown in Supplementary Information (uncropped blots). **e** KT-1 cells were starved overnight and then treated with IFNβ (10^4^ IU/mL) for 10 or 30 min, as indicated. After cell lysis, equal amounts of protein were immunoprecipitated (IP) with either PKCδ antibody or control normal rabbit immunoglobulin G (RIgG), as indicated. In vitro kinase assays to assess PKCδ activity were subsequently performed on the immunoprecipitates, using GST-ULK1 recombinant inactive protein as an exogenous substrate. (*Top panel*) Autoradiography film demonstrating PKCδ-induced phosphorylation of ULK1 after IFNβ treatment. (*Bottom panels*) Immunoblots demonstrating total GST-ULK1 protein and total immunoprecipitated PKCδ expression used in each condition for the in vitro kinase assay. Note: a lane between ULK1 and RIgG immunoprecipitates was loaded with 1x loading dye for best separation between the wells. Kinase assay shown is representative of three independent experiments. *unspecific band. **f** cDNA expression plasmids encoding the indicated myc-ULK1 derivatives were used for transfections and recombinant proteins were immunoprecipitated (c-Myc-IP) from HEK293T cells, inactivated, and subjected to in vitro kinase assays using GST-PKCδ recombinant protein as kinase. (*Top panel*) Autoradiography film demonstrating PKCδ-induced phosphorylation of myc-ULK1s and autophosphorylation of GST-PKCδ, as indicated. (*Bottom panel*) Coomassie Blue staining depicts loading/molecular weight (MW) of myc-ULK1 constructs. EV, myc-tagged empty vector. Kinase assay shown is representative of two independent experiments. Source data are provided as a Source Data file.

At the outset, we sought out the IFN-regulated signals controlling engagement of ULK1. There are putative PKCδ phosphorylation sites in ULK1^22^ and, identified from our previous work, PKCδ activity is essential for type I IFN-induced phosphorylation of p38 MAPK and the subsequent transcription of ISGs^23,24^. This prompted us to investigate if PKCδ and ULK1 interact during activation of IFNAR in *JAK2*^V617F^-positive cells. In co-immunoprecipitation assays using two *JAK2*^V617F^-expressing leukemia cell lines, we found ULK1 associated with PKCδ, both before and after IFNα-treatment (Fig. 1a, b). This constitutive interaction between PKCδ and ULK1, however, was not *JAK2*^V617F^-dependent, as it was also detected in *JAK2*^V617F^-negative leukemia lines (Fig. 1c, d). These results raised the possibility that IFN could stimulate PKCδ-induced phosphorylation of ULK1. Thus, to determine whether ULK1 is a substrate for PKCδ associated with type I IFN signaling, in vitro kinase assays were conducted on anti-PKCδ immunoprecipitates, using inactive GST-ULK1 as an exogenous substrate. Our results revealed that PKCδ phosphorylates ULK1 in a type I IFN-dependent manner (Fig. 1e).

**Fig. 2:**
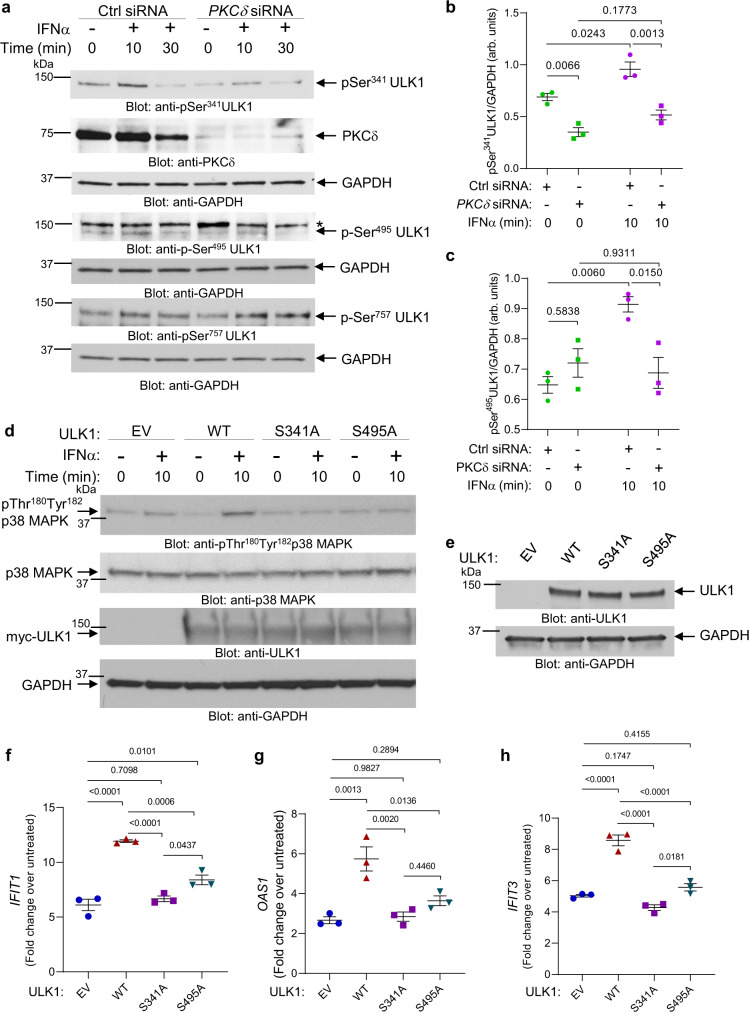
Type I IFN-induced PKCδ-dependent phosphorylation of ULK1 on serine residues 341 and 495 is required for ULK1-mediated activation of p38 MAPK and consequent expression of ISGs. **a** U937 cells were transfected with either control siRNA or PKCδ siRNA. 24 h later, cells were starved overnight (cultured in RPMI medium without FBS) and then were either left untreated or were treated with IFNα (10^4^ IU/mL) for 10 or 30 min, as indicated. Immunoblotting analysis was performed for the indicated proteins. Blots are representative of three independent experiments. *unspecific band. **b**, **c** Bands for the indicated proteins were scanned and quantified for untreated and 10 min IFNα-treated samples by densitometry, using ImageJ software. Quantified data are means ± SEM of pULK1/GAPDH from three independent experiments. Adjusted *p*-values are reported by two-way ANOVA followed by Tukey’s multiple comparisons test to assess the differences in ULK1 phosphorylation on Ser341 and Ser495 with treatment (untreated vs. IFNα), siRNA (Ctrl siRNA vs. PKCδ siRNA) and their interaction as predictors. arb. units, arbitrary units. **d** Immunoblotting analysis of p-p38 MAPK in lysates from *Ulk1/2*^−/−^ MEFs transfected with myc-tagged empty vector (EV), ULK1 WT, ULK1^S341A^ or ULK1^S495A^ plasmids treated with IFNα (10^4^ IU/mL) for 10 min, as indicated. Blots are representative of three independent experiments. **e**–**h** *ULK1* KO KT-1 cells were transfected with myc-tagged empty vector (EV), ULK1 WT, ULK1^S341A^ or ULK1^S495A^ plasmids. **e** Immunoblotting analysis of ULK1 in lysates from *ULK1* KO KT-1 transfected cells, as indicated. Blots are representative of three independent experiments. **f**–**h** qRT-PCR analysis of (**f**) *IFIT1*, (**g**) *OAS1*, and (**h**) *IFIT3* in *ULK1* KO KT-1 transfected cells treated for 6 h with IFNα (5000 IU/mL). Shown is mRNA expression fold change over respective untreated *ULK1* KO KT-1 transfected cells. Data are means ± SEM from three independent experiments. Adjusted *p*-values are reported by one-way ANOVA followed by Tukey’s multiple comparisons test. See also Supplementary Figs. 1–3. Source data are provided as a Source Data file.

**Fig. 3:**
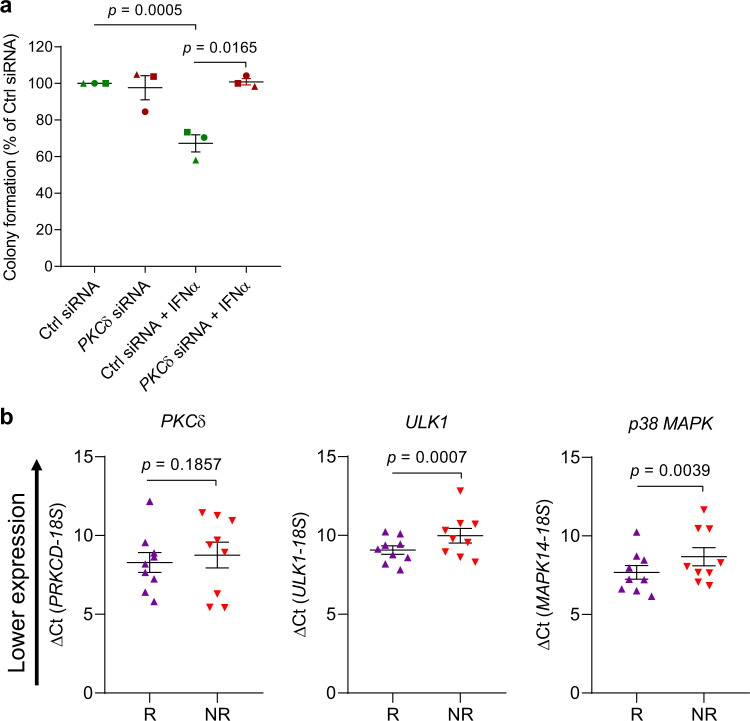
Correlation between PKCδ, ULK1 and p38 MAPK expression and responsiveness to IFN treatment in primary MPN cells. **a** Peripheral blood mononuclear cells from patients with PV were transfected with either control siRNA (Ctrl siRNA) or *PKCδ* siRNA, and the effects of IFNα treatment (10^3^ IU/mL) on malignant erythroid (BFU-E) colony formation were assessed by clonogenic assays in methylcellulose. Data are expressed as percent colony formation relative to control siRNA-transfected untreated cells (Ctrl siRNA) and represent means ± SEM of three independent experiments, using cells from three different individual patients with PV. In the graph, data for the same individual patient is represented by the same symbol for each experimental condition. Statistical analyses were performed using a linear mixed effects model, with % colony formation relative to the control group as the outcome, treatment (the three remaining groups) as the fixed effect, and subject as a random effect to account for within-subject correlation between multiple conditions. Kenward-Roger degrees of freedom adjustment was used, which improves performance when sample size is small. Pairwise group comparison tests were adjusted for multiple comparisons using Tukey’s method. Statistical significant *p*-values (two-sided) are reported. **b** Correlation between PKCδ (*PRKCD*), *ULK1*, or p38 MAPK (*MAPK14*) baseline mRNA expression and clinical response of PV or ET patients to PEG-IFNα treatment (clinical trial #NCT01259817)^7^ based on a simplified, two-group criteria: R, responders (*n* = 9); NR, non-responders (*n* = 9). The clinical characteristics of each patient are provided in Supplementary Table 1. *18S* gene expression was used as a reference gene. Data are expressed as ΔCt value for each gene, respectively (means ± SEM are shown). Statistical analyses were performed using linear mixed effects models^77^, with Ct as the outcome variable, and target (18S or one of PKCδ, ULK1 or MAPK14 genes), responder status (yes vs. no), and their interaction as fixed effects, and subject as the random effect to account for the correlation between within-subject replicates. Kenward-Roger degrees of freedom adjustment, which improves performance when sample size is small, was used. *p*-values (two-sided) are reported. Source data are provided as a Source Data file.

As previous studies using liquid chromatography (LC) and tandem mass spectrometry (MS/MS) have identified Ser341, Ser467 and Ser495 as the putative PKCδ phosphorylation sites in ULK1^22^, we next generated single serine-to-alanine (S/A) ULK1 mutants (S341A, S467A and S495A) and a triple (SSS/AAA) ULK1 mutant (S341/467/495 A). ULK1 WT and Ser-to-Ala ULK1 mutants were overexpressed in HEK293T cells, immunoprecipitated from cell lysates, inactivated, and used as substrates for purified active GST-PKCδ in the presence of ^32^P-ATP in kinase assays (Fig. 1f). While replacement of Ser467 by Ala did not result in an obvious alteration of phosphorylation, mutation of Ser341 to Ala (S341A) and Ser495 to Ala (S495A) reduced phosphorylation of ULK1 by PKCδ (Fig. 1f). Additionally, this reduction was more evident for the triple mutant (SSS/AAA) ULK1 (Fig. 1f). These results suggest that PKCδ directly phosphorylates ULK1 on both Ser341 and Ser495 residues (Fig. 1f), which prompted us to generate two phospho-specific ULK1 antibodies, one recognizing ULK1 phosphorylated on Ser^341^ and the other recognizing ULK1 phosphorylated on Ser^495^. We confirmed the specificity of the antibodies using *Ulk1/2*^−/−^ mouse embryonic fibroblasts (MEFs) transfected with myc-tagged empty vector, ULK1 WT, ULK1 S341A and ULK1 S495A mutants (Supplementary Fig. 1a, b). The p-Ser^341^ULK1 antibody gave a strong signal in the *Ulk1/2*^−/−^ cells transfected with both ULK1 WT and ULK1 S495A mutant, but not in cells transfected with empty vector or the ULK1 S341A mutant. Likewise, the p-Ser^495^ULK1 antibody gave a strong signal in the *Ulk1/2*^−/−^ cells transfected with both ULK1 WT and ULK1 S341A mutant, but not in the cells transfected with empty vector or ULK1 S495A mutant, confirming the specificity of these antibodies (Supplementary Fig. 1a, b). Next, using *Ulk1/2*^+/+^ MEFs, we provide evidence that phosphorylation of ULK1 on both Ser341 and Ser495 is induced by IFNα treatment (Supplementary Fig. 2). Moreover, using siRNA-mediated knockdown of PKCδ in U937 cells, we show that PKCδ expression is required for IFNα-mediated phosphorylation of ULK1 on Ser341 and Ser495, but not on Ser757, an AKT/mTOR phosphorylation site^21^ (Fig. 2a–c). As ULK1 is required for IFN-mediated activation of p38 MAPK, leading to transcription of ISGs^21^, we next sought to determine whether phosphorylation of ULK1 on Ser341 and Ser495 residues is essential for IFN-induced activation of p38 MAPK. To this end, we overexpressed myc-tagged empty vector, ULK1 WT, ULK1 S341A and ULK1 S495A in *Ulk1/2*^−/−^ MEFs. These cells were then treated with IFNα and the phosphorylation of p38 MAPK was assessed. ULK1 WT, but not the ULK1 S/A mutants, could rescue IFNα-induced activation of p38 MAPK (Fig. 2d). Additionally, we overexpressed myc-tagged empty vector, ULK1 WT, ULK1 S341A and ULK1 S495A in *ULK1* KO KT-1 cells. ULK1 protein expression or its absence was confirmed by anti-ULK1 immunoblotting (Supplementary Fig. 3 and Fig. 2e). The different transfected cells were then either left untreated or were treated with IFNα for 6 h and the mRNA expression of ISGs was evaluated by qRT-PCR analysis. In contrast with ULK1 S341A and ULK1 S495A, expression of the ULK1 WT substantially increased the IFNα-induced expression of *IFIT1*, *OAS1* and *IFIT3* compared to empty vector-transfected *ULK1* KO KT-1 cells (Fig. 2f–h). Moreover, siRNA-mediated targeted inhibition of PKCδ expression reversed the suppressive effects of IFNα on primitive malignant erythroid precursors (Fig. 3a). Thus, as previously shown for ULK1^21^ and p38 MAPK^25^, PKCδ engagement by activation of IFNAR is essential for the suppressive effects of IFNα on primary malignant PV progenitors, in vitro. Taken together these results provide evidence of previously unknown signaling events, in which activation of IFNAR controls PKCδ-mediated ULK1 phosphorylation on Ser341 and Ser495 and that ULK1 phosphorylation at these sites is required for downstream activation of p38 MAPK and transcription of ISGs.

To investigate whether constitutive levels of elements of this pathway may therefore discern responsiveness to IFN treatment, we examined mRNA expression levels of PKCδ, ULK1 and p38 MAPK in peripheral blood or bone marrow mononuclear cells from patients enrolled in the Myeloproliferative Disorders Research Consortium (MPD-RC)-111 study who received PEG-IFN-α2a (Pegasys) (clinical trial #NCT01259817)^7^ (Supplementary Table 1). Patients expressing higher pre-treatment mRNA levels of ULK1 and p38 MAPK were more likely to respond to PEG-IFN-α2a therapy (Fig. 3b), from which we infer a key role for this pathway in the anti-neoplastic effects of IFN in vivo. Future prospective clinical studies will be required to fully validate these findings and examine the potential of ULK1 and p38 MAPK as biomarkers of IFN-responsiveness.

### ROCK1 and ROCK2 are overexpressed in MPN patients and interact with ULK1

**Fig. 4:**
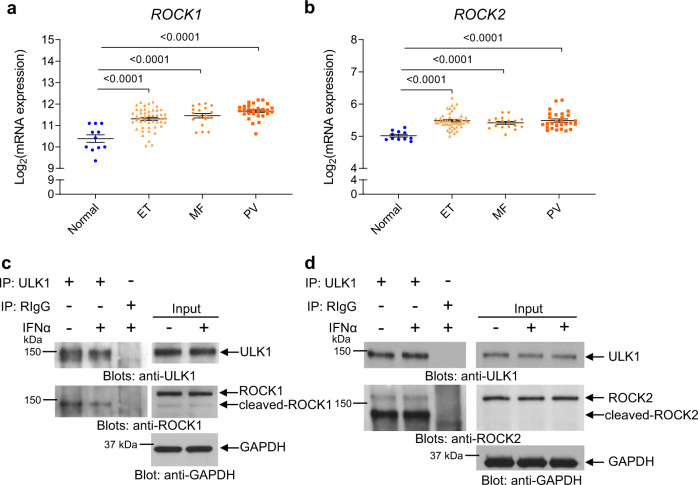
ROCK1 and ROCK2 interact with ULK1 in *JAK2*^V617F^-positive cells and are overexpressed in MPN patients. **a**, **b** Scatter dot plots of *ROCK1* and *ROCK2* expression in healthy individuals (normal, *n* = 11) and patients with ET (*n* = 47), MF (*n* = 18) and PV (*n* = 28). Data extracted from GSE54646^72^. Shown are means ± SEM of Log2 mRNA expression. Statistical analyses were performed using one-way ANOVA followed by Dunnett’s multiple comparisons test. Adjusted *p*-values are reported. **c**, **d** Immunoblots for the indicated proteins in total cell lysates (input) or in anti-ULK1 or RIgG (negative control) antibody immunoprecipitates (IP) from *JAK2*^V617F^-positive HEL cells left untreated or treated with IFNα (10^4^ IU/mL) for 10 min, as indicated. Blots are representative of three independent experiments. RIgG, normal Rabbit Immunoglobulin G. IP samples and corresponding total cell lysates (input) were resolved and blotted from the same gel as shown in Supplementary Information (uncropped blots). See also Supplementary Figs. 4 and 5. Source data are provided as a Source Data file.

Next, to identify binding partners of ULK1 in cells of MPN origin, we performed nano-LC MS/MS analysis of endogenous protein-ULK1 complexes isolated from untreated and IFNα-treated *JAK2*^V617F^-expressing HEL cells. The data indicate that ULK1 potentially interacts with 40 proteins in untreated cells, and with 38 proteins after IFNα treatment, 33 of which are identical in untreated and treated cells and 5 exclusively interact with ULK1 following IFN treatment (Supplementary Fig. 4, ProteomeXchange identifier PXD021748.). Among these five, is ROCK1 (Supplementary Fig. 4), a protein involved in promoting survival of some malignant hematopoietic cells^26,27^. We provide evidence that expression of both *ROCK1* and, its related isoform, *ROCK2*^28^, is increased in peripheral blood neutrophils from ET, PMF, and PV patients compared to age-matched healthy donors (Fig. 4a, b). Moreover, we used the DepMap Portal (https://depmap.org/portal/) to assess the relative protein expression of ROCK1 and ROCK2 in leukemia and other types of cancer cell lines (Supplementary Fig. 5). Interestingly, ROCK1 expression is higher in leukemia and lymphoma cell lines, compared to other types of cancer cell lines, whereas ROCK2 expression is the highest in colon cancer cells (Supplementary Fig. 5). Using co-immunoprecipitation followed by immunoblotting analyses, we found that ULK1 interacts preferentially with the cleaved/active forms of both ROCK1 and ROCK2 proteins^28^ in *JAK2*^V617F^-expressing cells (Fig. 4c, d).

**Fig. 5:**
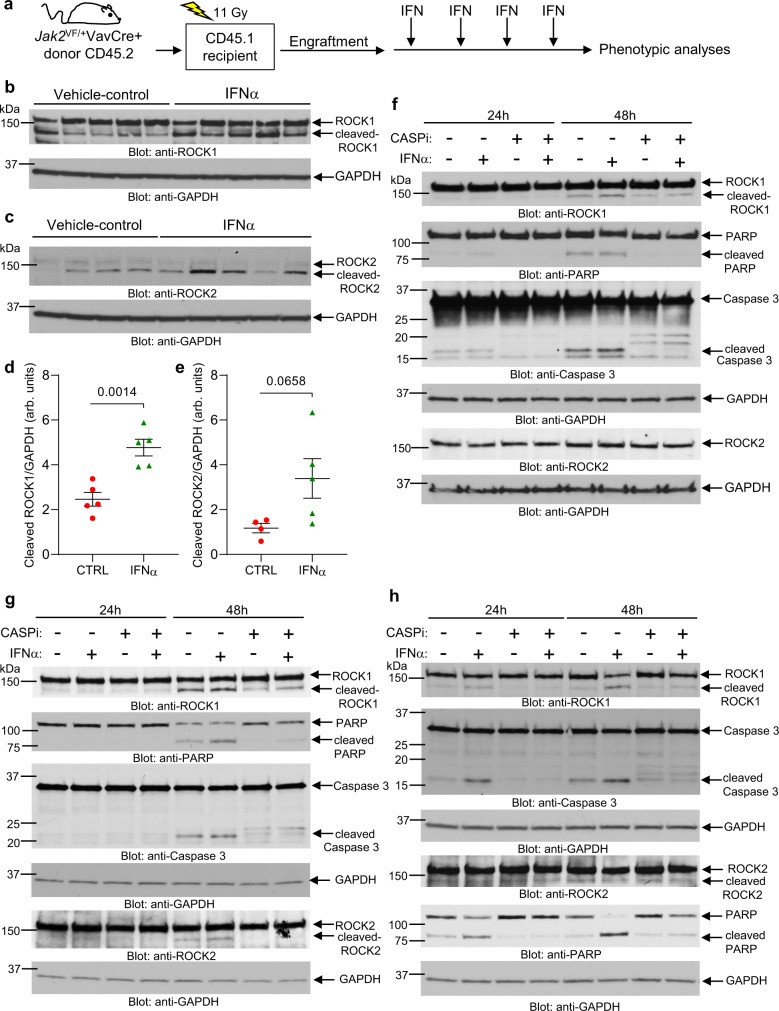
IFNα treatment induces cleavage/activation of ROCK1 and ROCK2 in a mouse model of MPN and in hematopoietic malignant cells in vitro. **a**–**e** CD45.1 mice were transplanted with *Jak2*^V617F^ KI bone marrow cells isolated from *JAK2*^V617F/+^VavCre+ CD45.2 donor mice. Five weeks after transplantation and confirmation of engraftment (see Supplementary Fig. 6a–f), CD45.1 recipient mice were treated with murine PEG-IFNα once a week (600 ng/mouse, subcutaneous injection) for 4 weeks or vehicle (PBS). **a** Schematic illustration of the MPN mouse model and treatment regimen performed before phenotypic analyses. **b**, **c** Western blot analysis of ROCK1 and ROCK2 in mononuclear bone marrow cells isolated 4 weeks after initiation of vehicle-control treatment (*n* = 5 for ROCK1, *n* = 4 for ROCK2) or PEG-IFNα treatment (*n* = 5 for ROCK1 and ROCK2) of CD45.1 recipient mice. **d**, **e** Bands for cleaved-ROCK1, cleaved-ROCK2 and respective GAPDH shown in **b** and **c** were quantified by densitometry using ImageJ software. Quantified data are means ± SEM of cleaved ROCK/GAPDH and each data point represents data for one individual mouse. Two-sample two-tailed *t*-test was performed to calculate differences in cleaved ROCK1/2 between the two treatment groups. *p*-values are reported. arb. units, arbitrary units. See also Supplementary Fig. 6g–j. **f**–**h** Immunoblotting analysis of the indicated proteins in lysates from (**f**) HEL, (**g**) U937, and (**h**) KT-1 cells either left untreated or treated with IFNα (1000 IU/mL) and/or the caspase inhibitor (CASPi) Z-VAD-FMK (20 μM) for 24 or 48 h, as indicated. Blots are representative of two independent experiments for each cell line. Source data are provided as a Source Data file.

**Fig. 6:**
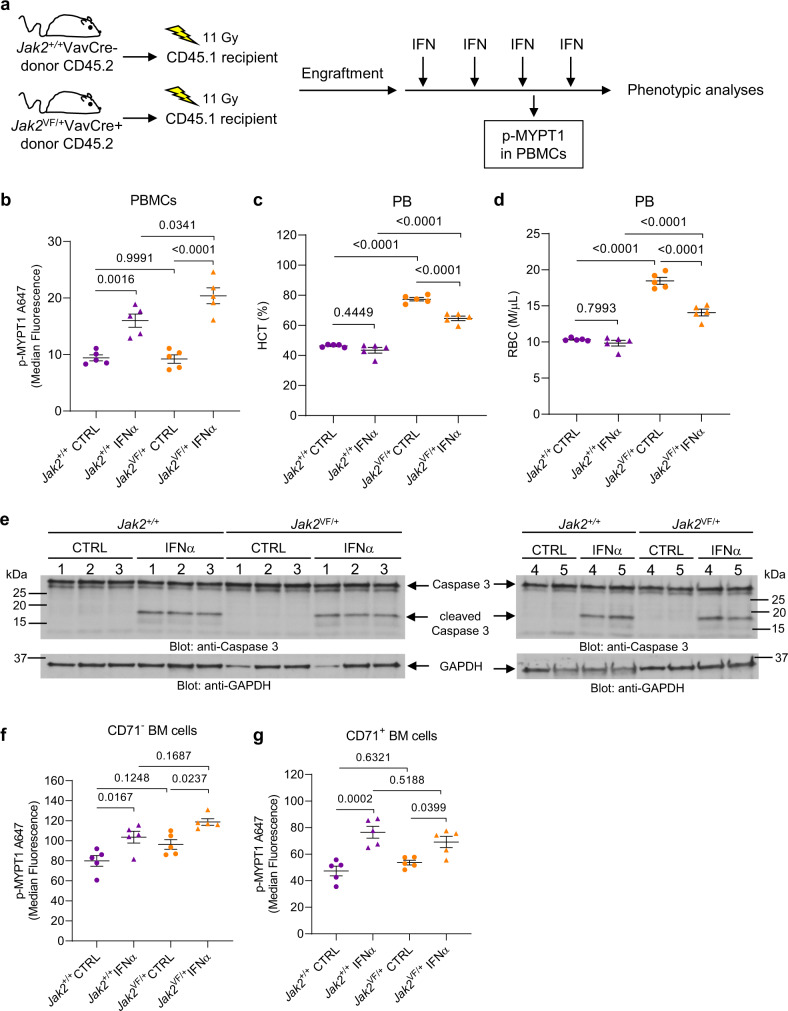
PEG-IFNα treatment induces cleavage/activation of caspase 3 and phosphorylation of ROCK1/2 downstream target MYPT1 in *Jak2*^+/+^ and *Jak2*^V617F/+^ hematopoietic cells in vivo. **a**–**g** CD45.1 mice were transplanted with either *Jak2*^+/+^ or *Jak2*^V617F/+^ KI bone marrow cells isolated from *Jak2*^+/+^VavCre- CD45.2 donor mice or *Jak2*^V617F/+^VavCre+ CD45.2 donor mice, respectively. Three weeks after transplantation and confirmation of engraftment (see Supplementary Fig. 7a–e), CD45.1 recipient mice were either treated with murine PEG-IFNα once a week (600 ng/mouse, subcutaneous injection) for 4 weeks or vehicle (PBS). **a** Schematic illustration of the in vivo models and treatment regimen performed before phenotypic analyses. Gy: gray units of ionizing radiation dose. **b** Scatter dot plots show median fluorescence of phosphorylated MYPT1 (p-MYPT1 A647) in *Jak2*^+/+^ (purple) or *Jak2*^V617F/+^ (orange) peripheral blood mononuclear cells (PBMCs) collected 24 h after the third dose of PEG-IFNα (triangles: *Jak2*^+/+^ IFNα *n* = 5 and *Jak2*^VF/+^ IFNα *n* = 5) or vehicle (circles: *Jak2*^+/+^ CTRL *n* = 5 and *Jak2*^VF/+^ CTRL *n* = 5). Data were assessed by flow cytometry and the gating strategy used is shown in Supplementary Fig. 7h. **c**, **d** Scatter dot plots show (**c**) hematocrit (HCT) and (**d**) red blood cell (RBC) counts of recipients from vehicle-control (*Jak2*^+/+^ CTRL *n* = 5 and *Jak2*^VF/+^ CTRL *n* = 5) and PEG-IFNα (*Jak2*^+/+^ IFNα *n* = 5 and *Jak2*^VF/+^ IFNα *n* = 5) groups after four doses of PBS (vehicle-control) or PEG-IFNα treatment. Data shown are means ± SEM. **e** Immunoblotting analysis of caspase 3 in lysates from bone marrow mononuclear cells (BMMCs) isolated from CD45.1 recipient mice 24 h after the fourth dose of PEG-IFNα (*Jak2*^+/+^ IFNα *n* = 5 and *Jak2*^VF/+^ IFNα *n* = 5) or vehicle (*Jak2*^+/+^ CTRL *n* = 5 and *Jak2*^VF/+^ CTRL *n* = 5), as indicated. **f**, **g** Scatter dot plots show median fluorescence of phosphorylated MYPT1 (p-MYPT1 A647) in *Jak2*^+/+^ or *Jak2*^V617F/+^ (**f**) CD71 negative (CD71-) and (**g**) CD71-positive (CD71+) BMMCs isolated from CD45.1 recipient mice 24 h after the fourth dose of PEG-IFNα (*Jak2*^+/+^ IFNα *n* = 5 and *Jak2*^VF/+^ IFNα *n* = 5) or vehicle (*Jak2*^+/+^ CTRL *n* = 5 and *Jak2*^VF/+^ CTRL *n* = 5). Data were assessed by flow cytometry and the gating strategy used is shown in Supplementary Fig. 7i. **b**–**d**, **f**, and **g** We used a two-way ANOVA to analyze the differences in **b**, **f**, and **g** p-MYPT1 median fluorescence, **c** percentage of HCT and **d** RBC counts with treatment (CTRL vs. IFNα), mutation (*Jak2*^+/+^ vs. *Jak2*^VF/+^) and their interaction as predictors. **b** The increase of MYPT1 phosphorylation induced by IFNα in *Jak2*^+/+^ PBMCs was smaller than in *Jak2*^VF/+^ PBMCs (*p* = 0.0382, interaction term). **c** The decrease of HCT percentage induced by IFNα in *Jak2*^+/+^ recipient mice was smaller than in *Jak2*^VF/+^ recipient mice (*p* = 0.0025, interaction term). **d** The decrease of RBC levels induced by IFNα in *Jak2*^+/+^ recipient mice was smaller than in *Jak2*^VF/+^ recipient mice (*p* = 0.0001, interaction term). **f**, **g** The difference in the increase of MYPT1 phosphorylation induced by IFNα in *Jak2*^+/+^ vs. *Jak2*^VF/+^ BM cells is not statistically significant (*p* > 0.05). Model-based pairwise comparisons were performed, and *p*-values were adjusted for multiple comparisons using Tukey’s method. *p*-values are reported. **b**–**g** Each data point or lane number represents data from an individual mouse. Results are representative of two independent in vivo studies. Source data are provided as a Source Data file.

Caspases mediate cleavage and subsequent activation of ROCK1/2^28^ and type I IFNs promote apoptosis through activation of caspases^29^, prompting us to examine whether IFNα treatment could induce cleavage/activation of ROCK1/2, in vivo. For these studies, we used a conditional *Jak2*^V617F^ knock-in (KI) MPN mouse model^30^ (Fig. 5a). As expected, 5 weeks after transplantation of *Jak2*^V617F^ KI bone marrow cells collected from *Jak2*^V617F/+^VavCre+ CD45.2 donor mice, a robust PV-like MPN phenotype developed in CD45.1 recipient mice (Supplementary Fig. 6a–f). Murine PEG-IFNα treatment was then initiated once a week for 4 weeks (Fig. 5a). As seen in previous MPN mouse model studies using IFNα^31–33^, PEG-IFNα treatment significantly reduced hematocrit (HCT) and red blood cell (RBC) levels in these mice (Supplementary Fig. 6g–j). Notably, PEG-IFNα treatment increased the cleavage of both ROCK1 and ROCK2 proteins in bone marrow mononuclear cells isolated from CD45.1 recipient mice (Fig. 5b–e). Additionally, we provide evidence that IFNα treatment induces activation/cleavage of caspase 3, PARP and ROCK proteins in both *JAK2*^V617F^-positive HEL cells and in *JAK2*^V617F^-negative U937 and KT-1 cells, in vitro, and these effects are repressed by co-treatment with a pan-caspase inhibitor, Z-VAD-FMK (Fig. 5f–h). In further studies, to determine whether PEG-IFNα treatment induces activation/cleavage of caspase 3 and activation of ROCK proteins in both *Jak2*^+/+^ (wild-type) and *Jak2*^V617F/+^ hematopoietic cells in vivo, we transplanted CD45.1 recipient mice with either *Jak2*^+/+^ or *Jak2*^V617F/+^ KI bone marrow cells collected from *Jak2*^+/+^VavCre- and *Jak2*^V617F/+^VavCre+ CD45.2 donor mice, respectively (Fig. 6a). Three weeks after transplantation, engraftment of *Jak2*^+/+^ and *Jak2*^V617F/+^ CD45.2+ cells in CD45.1 recipient mice was confirmed (Supplementary Fig. 7a) and a PV-like MPN phenotype developed in *Jak2*^V617F/+^, but not in *Jak2*^+/+^, recipient mice (Supplementary Fig. 7b–e). Murine PEG-IFNα treatment was then initiated once a week for 4 weeks (Fig. 6a). At week 3, 24 h post-IFNα treatment we collected peripheral blood and isolated mononuclear cells from the recipient mice (Fig. 6a, p-MYPT1 in peripheral blood mononuclear cells (PBMCs)). In both *Jak2*^+/+^ and *Jak2*^V617F/+^ recipient mice, PEG-IFNα treatment increased phosphorylation of the ROCK1/2 downstream target myosin phosphatase target subunit 1 (MYPT1)^28^, compared to vehicle-control-treated mice (Fig. 6b), consistent with an IFNα-induced increase in ROCK1/2 kinase activity. Importantly, IFNα-induced phosphorylation of p-MYPT1 was greater in *Jak2*^V617F/+^ compared to *Jak2*^+/+^ recipient mice (*p* = 0.0382). Additionally, PEG-IFNα treatment decreased hematocrit and red blood cell levels in *Jak2*^V617F/+^, but not in *Jak2*^+/+^, recipient mice (Fig. 6c, d and Supplementary Fig. 7f, g), consistent with earlier reports that IFNα treatment targets *JAK2*^V617F^-mutant over non-malignant hematopoietic cells^32^. Twenty-four hours after the fourth dose of PEG-IFNα, mononuclear bone marrow cells from recipient mice were isolated and we evaluated cleavage/activation of caspase 3 by immunoblotting and phosphorylation of MYPT1 by flow cytometry analyses. The data show that PEG-IFNα treatment induces cleavage of caspase 3 (Fig. 6e) and phosphorylation of MYPT1 (Fig. 6f, g) in both *Jak2*^+/+^ and *Jak2*^V617F/+^ bone marrow cells. These results were consistent with the analyses performed using bone marrow cells isolated from PEG-IFNα-treated wild-type C57BL/6 J mice (Supplementary Fig. 8). Together these results suggest that IFNα treatment induces caspase-dependent ROCK1/2 activation in normal and malignant hematopoietic cells, suggesting a potential role for ROCK1/2 in regulation of IFN-mediated biological responses.

### Inhibition of ROCK1/2 enhances IFNα-dependent anti-MPN responses

**Fig. 7:**
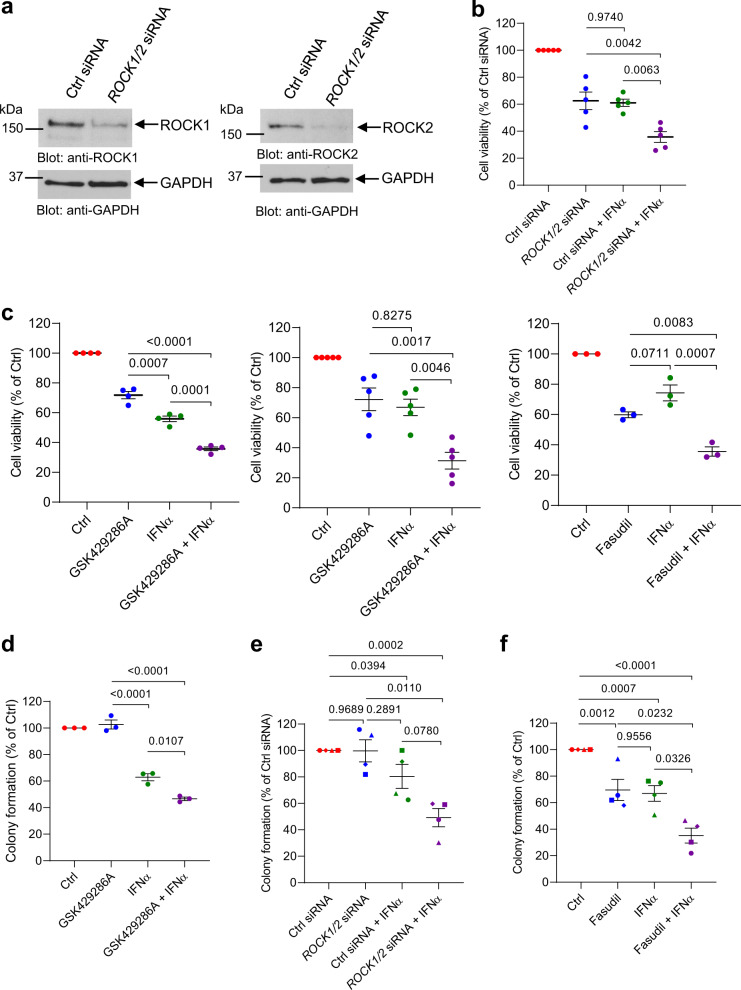
Inhibition of ROCK1 and ROCK2 enhances IFNα-induced anti-neoplastic responses in MPN cells. **a** Immunoblotting analysis of ROCK1 and ROCK2 in lysates from control (Ctrl) siRNA or ROCK1 and ROCK2 (*ROCK1/2*) siRNA-transfected *JAK2*^V617F^-positive SET-2 cells. **b** Proliferation and cell viability of control (Ctrl) siRNA or ROCK1 and ROCK2 (*ROCK1/2*) siRNA-transfected *JAK2*^V617F^-positive SET-2 cells either left untreated, or treated with IFNα was measured using a WST assay. Data are expressed as percent cell viability relative to Ctrl siRNA-transfected untreated cells (Ctrl siRNA), and represent means ± SEM of five independent experiments. Each symbol on the graphs represents an independent biological replicate. **c** Proliferation of *JAK2*^V617F^-positive (*left panel*) SET-2 or (*middle and right panels*) HEL cells treated with ROCK1/2 inhibitor (GSK429286A or Fasudil) and/or IFNα vs. DMSO-vehicle control (Ctrl) was measured using a WST assay. Each symbol on the graphs represents an independent biological replicate. Data are expressed as percent cell viability relative to Ctrl-treated cells and represent means ± SEM of four, five and three independent experiments, as indicated. **d** Clonogenic capability of *JAK2*^V617F^-positive HEL cells treated with the ROCK1/2 inhibitor GSK429286A and/or IFNα vs. DMSO-vehicle control (Ctrl). Each symbol on the graphs represents an independent biological replicate. Data are expressed as percent colony formation relative to Ctrl-treated cells and represent means ± SEM of three independent experiments. **e** Clonogenic capability of Ctrl siRNA or *ROCK1/2* siRNA-transfected peripheral blood mononuclear cells from patients with PV, either left untreated or treated with IFNα. Data are expressed as percent colony formation relative to control siRNA-transfected untreated cells (Ctrl siRNA) and represent means ± SEM of four independent experiments, using cells from four different patients with PV. **f** Clonogenic capability of peripheral blood mononuclear cells from patients with PV treated with the ROCK1/2 inhibitor Fasudil and/or IFNα vs. DMSO-vehicle control (Ctrl). Data are expressed as percent colony formation relative to Ctrl-treated cells and represent means ± SEM of four independent experiments, as indicated, using cells from four different patients with PV. **e**, **f** Each data point on the graphs represents an independent biological replicate and data for an individual patient is represented by the same symbol for each experimental condition. **b**–**d** Data were normalized to the control group (Ctrl siRNA or Ctrl) and statistical analyses comparing the other three groups were performed using one-way ANOVA followed by Tukey’s multiple comparisons test. Adjusted *p*-values are reported. **e**, **f** Data were analyzed using linear mixed effects models, with % colony formation relative to the control group as the outcome, treatment (the three remaining groups) as the fixed effect, and subject as a random effect to account for within-subject correlation between multiple conditions. Kenward-Roger degrees of freedom adjustment was used, which improves performance when sample size is small. Pairwise group comparison tests were adjusted for multiple comparisons using Tukey’s method. *p*-values are reported. Source data are provided as a Source Data file.

First, we sought to determine whether ROCK1/2 expression is required for transcription of ISGs. siRNA-mediated knockdown of *ROCK1/2* (Supplementary Fig. 9a, b) did not enhance or inhibit IFNα-induced transcription of ISGs in *JAK2*^V617F^-positive HEL cells (Supplementary Fig. 9c–f). To determine the effects of ROCK1/2 activation on IFN anti-neoplastic responses, we evaluated the effects of genetic or drug-targeted inhibition of ROCK1/2 in *JAK2*^V617F^-positive cells, alone or in combination with IFNα. siRNA-mediated inhibition of *ROCK1/2* expression (Fig. 7a) in combination with IFNα treatment significantly decreased cellular viability of SET-2 cells, compared to either *ROCK1/2* knockdown or IFNα treatment alone (Fig. 7b). Similar effects were observed in SET-2 (Fig. 7c, *left panel*) and HEL (Fig. 7c, *middle and right panel*) cells treated with the ROCK1/2 inhibitors GSK429286A or Fasudil in combination with IFNα treatment, compared to treatment with either inhibitor alone. Moreover, GSK429286A in combination with IFNα treatment significantly reduced the clonogenic capacity of HEL cells, compared to either inhibitor alone (Fig. 7d). Importantly, siRNA-mediated knockdown of *ROCK1/2* expression or drug-targeted inhibition of ROCK1/2 activity with Fasudil enhanced the suppressive effects of IFNα on primitive malignant erythroid precursors from PV patients who responded poorly to IFN treatment (Fig. 7e, f).

**Fig. 8:**
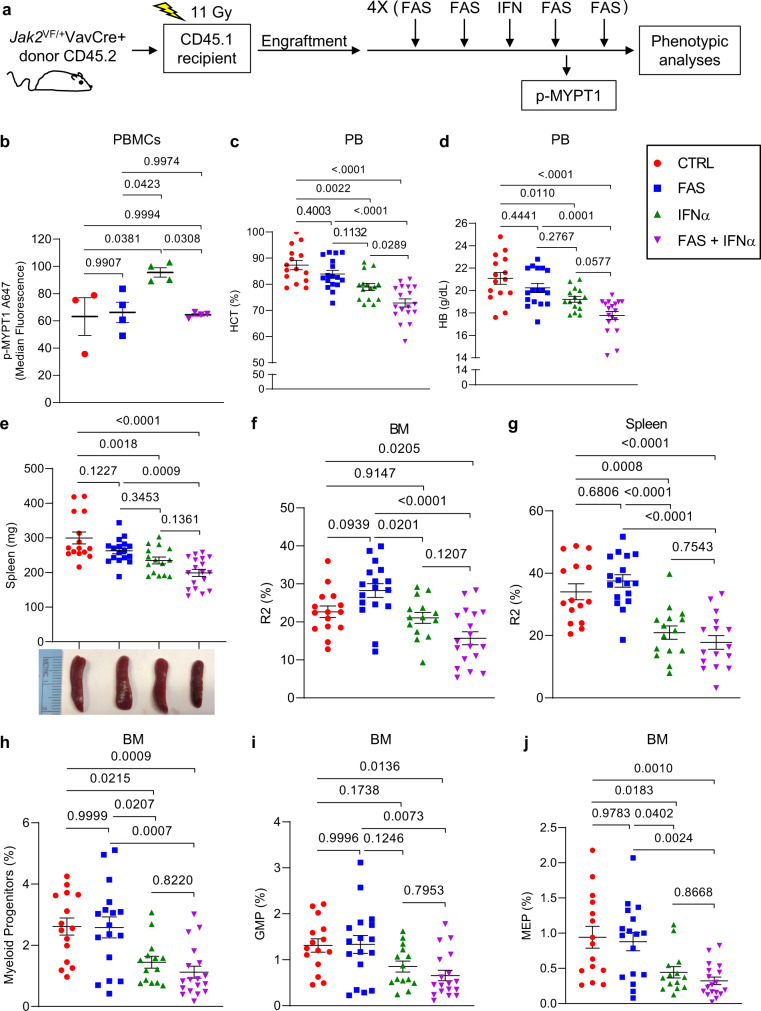
The ROCK1/2 inhibitor, Fasudil, blocks PEG-IFNα induced ROCK1/2 activation and enhances PEG-IFNα effects in a MPN mouse model. **a**–**j** CD45.1 mice were transplanted with *Jak2*^V617F/+^ KI bone marrow cells isolated from *Jak2*^V617F/+^VavCre+ CD45.2 donor mice. Five weeks after transplantation and confirmation of engraftment (see Supplementary Fig. 10a–e), CD45.1 recipient mice were treated with vehicles (PBS, CTRL), or PEG-IFNα (IFNα, 600 ng/mouse, subcutaneous injection) once a week, and/or Fasudil (FAS, 25 mg/kg, intraperitoneal injection) four times per week for 4 weeks. **a** Schematic illustration of the MPN mouse model, treatment regimen per week, and analyses performed. Gy: gray units of ionizing radiation dose. **b** Scatter dot plots show median fluorescence of phosphorylated MYPT1 (p-MYPT1 A647) in peripheral blood mononuclear cells collected 26 h after the third dose of PEG-IFNα and 2 h after the eleventh dose of Fasudil and/or respective vehicles (CTRL, *n* = 3; FAS, *n* = 4; IFNα, *n* = 4; FAS + IFNα, *n* = 4). Data were assessed by flow cytometry and the gating strategy used is shown in Supplementary Fig. 7h. Results are representative of two independent experiments. **c**–**j** Scatter dot plots show effects of Fasudil (FAS) and/or PEG-IFNα (IFNα) treatments on (**c**) hematocrit (HCT) percentage and (**d**) hemoglobin (HB) levels in peripheral blood, (**e**) spleen weight (images are representative of spleen size per treatment group), **f**–**g** percentage of Ter119^high^CD71^high^ basophilic erythroblasts (R2) in **f** bone marrow (BM) and **g** spleen, and on the **h**–**j** percentage of bone marrow **h** myeloid progenitors, **i** granulocyte-monocyte progenitors (GMP) and **j** megakaryocyte-erythroid progenitors (MEP). **c**–**j** Data shown are means ± SEM pooled from four independent studies (CTRL *n* = 15, FAS *n* = 17, IFNα *n* = 15, FAS + IFNα *n* = 18); each independent study performed using 3–6 mice per treatment group. The flow cytometry gating strategy used is shown in Supplementary Figs. S11a and S11f. **b**–**j** Statistical analyses were performed using one-way ANOVA followed by Tukey’s multiple comparisons test. Adjusted *p*-values are reported. See also Supplementary Figs. 10 and 11. Source data are provided as a Source Data file.

**Fig. 9:**
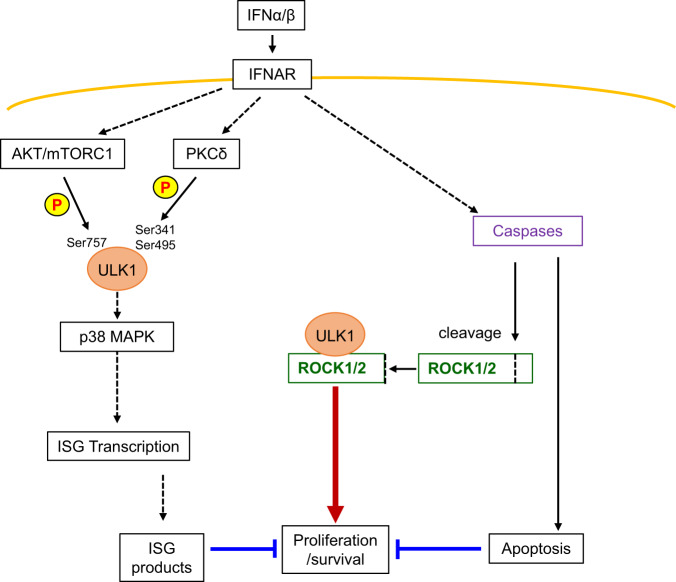
Schematic illustration of the proposed signaling feedback circuit activated by type I IFNs and regulation of IFN-mediated anti-neoplastic responses. Type I IFNs bind their transmembrane receptor (IFNAR) activating AKT/mTORC1-dependent phosphorylation of ULK1 on Ser757, which inhibits ULK1 engagement into autophagy-related pathways^21^. In parallel, IFN-mediated downstream activation of PKCδ kinase activity induces ULK1 phosphorylation on Ser341 and Ser495, required for downstream phosphorylation of p38 MAPK and transcription of ISGs, which inhibit proliferation and survival of MPN cells. Engagement of IFNAR also induces activation of caspases leading to apoptosis of MPN cells. However, caspase activation mediates cleavage/activation of ROCK1/2 proteins, found to bind ULK1, that drive pro-survival mechanisms in MPN cells, negatively regulating IFN anti-neoplastic effects.

Next, we examined the effects of targeting ROCK1/2 on the inhibitory effects of IFNα in our MPN mouse model. Five weeks after transplantation of CD45.1 recipient mice with *Jak2*^V617F^ KI bone marrow cells (Supplementary Fig. 10a–e), we treated mice for 4 weeks with the ROCK inhibitor Fasudil^34^ four times per week, with or without PEG-IFNα treatment given once a week (Fig. 8a). Mice tolerated single agent and combination drug treatments well, as monitored by body weight (Supplementary Fig. 10f). At week 3 after initiation of treatment, we collected peripheral blood and isolated mononuclear cells from the recipient mice 26 h post-IFNα treatment and 2 h post-Fasudil treatment or its vehicles (Fig. 8a, p-MYPT1). PEG-IFNα treatment increased phosphorylation of MYPT1 compared to vehicle-control and Fasudil only treated mice (Fig. 8b), and co-treatment with Fasudil and PEG-IFNα inhibited this increase in peripheral blood mononuclear cells (Fig. 8b). Four weeks post-treatment, further decreases in hematocrit (HCT) and hemoglobin (HB) were noticeable in the IFNα + Fasudil-treated mice, compared to mice that were treated with vehicle or either drug alone (Fig. 8c, d). Red blood cell counts were significantly decreased in both IFNα- and IFNα + Fasudil-treated mice compared to vehicle- and Fasudil-treated mice (Supplementary Fig. 10g). By contrast, no significant differences were observed in platelet levels among the four treatment groups (Supplementary Fig. 10h). Additionally, the reductions in spleen weight, percentage of bone marrow and spleen Ter119^high^CD71^high^ basophilic erythroblasts (R2), and bone marrow myeloid progenitors, granulocyte-monocyte progenitors (GMPs), and megakaryocyte-erythroid progenitors (MEPs) between vehicle-treated and IFNα + Fasudil-treated mice are greater than between vehicle-treated and IFNα-treated mice (Fig. 8e–j). No significant differences were observed between IFNα and IFNα + Fasudil-treated mice for bone marrow and spleen Ter119^med^CD71^high^ proerythroblasts (R1), Ter119^high^CD71^med^ polychromatophilic erythroblasts and Ter119^high^CD71^low^ orthochromatophilic erythroblasts (R3 + R4) and for bone marrow hematopoietic stem cells (Lin^-^Sca^+^Kit^+^, LSK) and common myeloid progenitors (CMP) (Supplementary Fig. 11). Taken together with the in vitro findings, these results suggest that IFNα treatment activates ROCK1/2 proteins in a negative feedback regulatory manner, triggering mechanisms that suppress its anti-neoplastic effects in MPNs (Fig. 9), and inhibition of activated ROCK1/2 proteins enhances the anti-neoplastic effects of IFNα in MPNs.

## Discussion

Over the past 4 decades, accumulating evidence has revealed that type I IFNs exert important anti-tumor effects and have a critical role in immune surveillance against the development of malignancies^3,6^. A precise understanding of the mechanisms of action of type I IFNs on both tumor and immune cells is critical, as this will provide the means to identify, in advance, patients who will respond to IFN treatment and thereby predict clinical responses. Moreover, this knowledge will lead to new therapeutic approaches and the development of combination treatments to enhance the action(s) of IFNs and/or overcome resistance mechanisms. It is well established that activation of IFNAR initiates signaling events mediated via the classical JAK/STAT pathway and many other “non-classical” pathways^6,35^. It is the concerted actions of these signaling pathways and their effectors that drive the biological outcomes of IFN treatment and, at the same time, control excessive responses^35^. We now identify PKCδ as a key initiator of the p38 MAPK pathway and demonstrate that PKCδ binds to and directly phosphorylates ULK1 on serine residues 341 and 495. We establish that phosphorylation of ULK1 on these sites is required for downstream engagement of p38 MAPK and transcription of ISGs. Activation of PKCδ is critical for the generation of IFN responses, as we provide evidence that PKCδ knockdown reverses the IFNα-mediated anti-clonogenic effects in PV patient primary cells. Using MPN patient samples collected prior to initiation of PEG-IFNα therapy, we observed that higher baseline expression levels of the downstream effectors of PKCδ, namely ULK1 and p38 MAPK, predict a better response to IFNα treatment. These findings suggest that ULK1 and p38 MAPK could potentially be used as part of a pool of biomarkers to predict response to IFNα-based therapies for MPN patients, but future large prospective clinical studies will be required to definitively address this.

In previous studies, PKCδ has been characterized as both a tumor-promoting and a tumor-suppressing kinase, dependent on the stimulus and cell-type context^36–39^. PKCδ promotes cellular apoptosis through both p38 MAPK-dependent and -independent mechanisms, induces cell cycle arrest and inhibits tumor growth and progression^39^. By contrast, PKCδ enhances the migration, invasion, and metastasis of some tumor cells^40–43^. Similarly, ULK1 has been shown to have opposing roles in cancer, again depending on the cellular context and stimulus^44^. In response to stress, such as starvation, ULK1 is phosphorylated on Ser555 by AMPK to induce autophagy^45^, shown to play tumor-promoting and tumor-suppressing functions in a context-dependent manner^46^. Additionally, other stimuli, such as IFNs, induce ULK1 engagement in autophagy-independent signaling events, controlling several biological functions^21,44,47–51^. Phosphorylation of ULK1 on Ser757 (autophagy-inhibitory site) is associated with shorter overall survival in non-small cell lung cancer patients^52^. In breast cancer patients, low levels of ULK1 correlate with lymph node metastasis and poor survival^53^. Hypoxia-induced expression of the kinase-dead ULK1 mutant increases lung metastasis in a breast cancer xenograft mouse model^54^ and, in another study, ULK1 was shown to phosphorylate Exo70 to inhibit breast cancer metastasis^55^. In contrast, a kinase-dead ULK1 mutant suppressed growth and metastasis and increased survival in a neuroblastoma xenograft mouse model^56^. We now report that activation of a PKCδ-ULK1-p38 MAPK signaling pathway is required for IFNα-induced anti-neoplastic effects in MPN patients.

Interestingly, in a recent study, the atypical PKC λ/ι isoform was found to directly phosphorylate ULK2, inhibiting its activity and promoting its degradation^57^. Inactivation/loss of PKCλ/ι was found to induce ULK2 activity, which directly activates the TBK1-STING-IRF3 pathway, leading to IFN production and anti-tumor responses^57^. In another study, the PKCδ isozyme, PKCα, was shown to induce phosphorylation of ULK1 on Ser423, reducing autolysosome formation and promoting ULK1 degradation^58^. Additional studies are required to interrogate the potential role of PKCδ-induced phosphorylation of ULK1 on Ser341 and Ser495 in the regulation of autophagy and IFN production.

Beyond defining the mechanism by which the ULK1/p38 MAPK pathway is engaged in PV cells, our studies have identified an important feedback mechanism that negatively regulates IFN-responses, that may be a therapeutic target. This involves ROCK1/2, shown to interact with ULK1. Importantly, we provide evidence that IFNα treatment induces cleavage/activation of ROCK1/2 in vitro and in vivo in both *JAK2*^+/+^ and *JAK2*^V617F/+^ hematopoietic cells. Since IFNα treatment does not affect hematocrit, hemoglobin, red blood cells and platelet levels in *Jak2*^+/+^ recipient mice, our results suggest that IFN-mediated activation of ROCK1/2 could represent a feedback mechanism to control the IFN effects on normal hematopoiesis. In contrast, we found that both ROCK isoforms are overexpressed in peripheral blood cells from MPN patients, compared to healthy individuals and genetic or pharmacological inhibition of ROCK1/2 enhanced IFNα-induced anti-neoplastic effects in primary MPN cells and in a MPN model in vivo. Our findings support a role for the IFN-mediated activation of ROCK1/2 as pro-tumorigenic in MPN cells. In other studies, ROCK1/2 have been shown to regulate actomyosin contractility and cytoskeleton assembly, important features for tumor progression, migration and invasion^59–62^. ROCK1 and ROCK2 have also been shown to have redundant roles in cell cycle progression and have been implicated in tumorigenesis^63^. Genetic deletion of not one, but both ROCK isoforms, blocked tumor formation in mouse models of non-small cell lung cancer and melanoma^63^. Recently, engagement of ROCK-myosin II signaling was identified as a mechanism of resistance to MAPK inhibitors in melanoma^64^. Furthermore, the ROCK inhibitor, Fasudil, has been shown to decrease disease burden in myeloproliferative disease (MPD) mouse models^27,65^ and combination of imatinib with either Fasudil or the ROCK inhibitor Y-27632 showed synergistic inhibition of CD34^+^ chronic myeloid progenitor cell growth^66^. To date, the ROCK inhibitors Fasudil and Ripasudil are approved in Japan for the treatment of cerebral vasospasm and glaucoma, respectively, and several ROCK inhibitors are being tested clinically for the treatment of different diseases, including cancer^34^.

Viewed altogether, our data identify an important IFNα-regulated negative feedback mechanism that leads to caspase-dependent cleavage/activation of ROCK1/2, which interacts with ULK1, and negatively regulates IFNα anti-neoplastic responses. These findings support future studies to evaluate the clinical potential of combinations of ROCK inhibitors with PEG-IFNα and/or the JAK inhibitor ruxolitinib^33^ for the treatment of MPN patients.

## Methods

### Patient-derived samples

Studies were approved by the Institutional Review Board of Northwestern University in accordance to the Declaration of Helsinki protocol. Peripheral blood (PB) was collected from patients with Polycythemia Vera (PV) after their informed consent and PBMCs were isolated following Histopaque density gradient separation (Sigma). PB and bone marrow (BM) MCs from patients with PV or ET were also obtained from the New York Blood Center bank, including samples collected at baseline from clinical trial # NCT01259817 after patients’ informed consent.

For the study correlating expression of genes of interest with clinical outcomes to IFN treatment, primary samples collected before treatment from 18 patients (9 responders and 9 non-responders) were randomly selected with the assistance of the MPN-RC Biostatistics Core C and provided by the MPN-RC Tissue Bank Core B for our analysis. MPN patients had been enrolled in the MPD-RC-111 study, which included patients with polycythemia vera (PV) and essential thrombocythemia (ET) that were refractory or intolerant to hydroxyurea therapy (clinical trial #NCT01259817), after providing informed consent. Fifteen patients were diagnosed as having PV while 3 patients had ET (9 males, 9 females, between ages 36-84 years). The clinical characteristics of each patient are provided in Supplementary Table 1. Responses were assessed after 12 months of treatment^7^ according to criteria established by the European LeukemiaNET. Follow-up information on responses was available after 24 months for some of the patients at the time of the qRT-PCR analysis. There were 9 responders and 9 non-responders analyzed. One patient had a partial response after 12 months, but was classified as a non-responder after 24 months of IFN treatment and was therefore included as a non-responder in Fig. 3b. A more detailed description of participants enrolled in the clinical trial #NCT01259817 and criteria for assessment of clinical response have been previously described^7^. The criteria for enrollment of patients in clinical trial #NCT01259817 have been previously described^7^. Samples for banking and for the in vitro colony studies were obtained from MPN patients seen for evaluation, follow-up, and/or treatment in the clinics of the respective institutions, after informed consent was obtained.

PBMCs and bone marrow mononuclear cells (BMMCs) were used to perform qRT-PCR analyses and PBMCs were used to perform hematopoietic cell progenitor assays.

### Animals

Mice were housed in a pathogen-free barrier facility at Northwestern University. The light cycle is 14:10, lights are on at 5  a.m., off at 7 p.m.. The room temperature is 72° Fahrenheit (F) +/– 2° (70–74 °F, 21.1–23.3° celsius) and room humidity ranges 30–70% (average is 50%). The animals are housed in ventilated animal holding racks with building supplied HEPA filter air directly to the ventilated racks. Supply air is provided via separate plenums from the exhausted cage air. Cage change cycle is cage bottoms changed out every 14 days unless excessively soiled and complete change out every 4 weeks. The animals are provided reverse osmosis water through an automatic watering system directly to the cage. Racks are flushed daily and checked daily to verify water availability. All animal studies were approved by the Northwestern University Institutional Animal Care and Use Committee (Protocol # IS00012751) and conducted in accordance with the Guidelines for the Care and Use of Laboratory Animals. Both female and male (6–12-week-old) *Jak2*^V617F/+^ conditional knock-in (KI) mice (CD45.2)^30^ (provided by B. L. Ebert and A. Mullally, Harvard Medical School) and Vav-Cre^+^ (CD45.2) (B6.Cg-Commd10^Tg(Vav1-icre)A2Kio^/J) (The Jackson Laboratory Stock, Stock No: 008610) mice were used for breeding to generate *Jak2*^+/+^VavCre^-^ and *Jak2*^V617F/+^VavCre^+^ donor mice. In all, 10–11-week-old female and male CD45.1 (B6.SJL-*Ptprc*^a^*Pepc*^b^/BoyJ) mice (The Jackson Laboratory, Stock No. 002014) were used as recipients and 6–10-week-old female and male *Jak2*^+/+^VavCre^-^ and *Jak2*^V617F/+^VavCre^+^ donor mice were used as donors for the transplant studies. Once engraftment and a PV-like phenotype (for the *Jak2*^V617F/+^VavCre^+^ recipients) was confirmed in recipient mice, these were randomized into treatment groups by hematocrit and/or hemoglobin levels. In all, 9–10-week-old female C57BL/6J mice (The Jackson Laboratory, Stock No: 000664) were used for the studies to assess the effects of IFNα treatment in wild-type mice in vivo. These mice were randomized into treatment groups by body weight.

### Cells and cell culture

HEL (TIB-180; ATCC), KT-1^67^ (RRID:CVCL_D200) and U937 (CRL-1593.2; ATCC) cells were grown in RPMI 1640 medium (Gibco) supplemented with 10% fetal bovine serum (FBS) and antibiotics. SET-2 cells (ACC-608; DSMZ) were grown in RPMI 1640 medium (Gibco) supplemented with 20% FBS and antibiotics. HEK293T cells (CRL-3216; ATCC) and the immortalized *Ulk1/2*^+/+^ and *Ulk1/2*^−/−^ MEFs^68^ provided by C. B. Thompson (Memorial Sloan-Kettering Cancer Center) were grown in DMEM medium (Gibco) supplemented with 10% FBS and antibiotics. All cell lines were frozen at low passage in liquid nitrogen and were kept in culture for no longer than ten-fifteen passages. All cells were cultured at 37 °C and 5% CO_2_. All cells were tested for mycoplasma contamination using a MycoAlert PLUS mycoplasma detection kit, following the manufacturer’s instructions (Lonza). Cell lines were tested by STR analyses and matched to the ATCC or DSMZ database every 6 months to 1 year.

### Generation of *ULK1* KO KT-1 cells using CRISPR-Cas9 technology

*ULK1* KO KT-1 cells were generated using Edit-R Lentiviral hEF1α-Blast-Cas9 Nuclease Plasmid DNA and Edit-R Human ULK1 Lentiviral sgRNA (no. CAS10138 and no. VSGH10142-246477203, GE Healthcare Dharmacon), as previously described^48^. Lack of ULK1 protein in *ULK1* KO KT-1 cells was confirmed by immunobloting analysis.

### Plasmids, transfections, and cell treatments

The pRK5/myc-hULK1 plasmid (a gift from Do-Hyung Kim; Addgene plasmid #31961; http://n2t.net/addgene:31961; RRID:Addgene_31961)^69^ was used to generate the single serine-to-alanine ULK1 mutants (S341A, S467A, and S495A) and a triple ULK1 mutant (S341/467/495A) plasmids, using site-directed mutagenesis, by BioInnovatise, Inc. Mutations were confirmed by DNA sequencing analyses using the following primer: 5’cgagaagaacaagacgttgg3’ and BLASTN program (https://blast.ncbi.nlm.nih.gov/Blast.cgi?PROGRAM=blastn&BLAST_SPEC=GeoBlast&PAGE_TYPE=BlastSearch). The pRK5 empty vector (EV) was made by digestion of pRK5/myc-hULK1 plasmid with SalI and NotI restriction enzymes to release ULK1 gene, followed by fill-in and ligation reactions by BioInnovatise, Inc. The EV was sequenced using the SP6 primer: 5’gatttaggtgacactatag3’. HEK293T cells were transfected with plasmid using Lipofectamine 2000 transfection reagent (Invitrogen), according to the manufacturer’s protocol and then used for immunoprecipitation of myc-tagged proteins. *Ulk1/2*^−/−^ MEFs were transfected with plasmid using TurboFect Transfection Reagent (Thermo Fisher Scientific), according to the manufacturer’s protocol. The next day, *Ulk1/2*^−/−^ cells were either left untreated or were treated with mouse IFNα (10 × 10^3^ IU/mL) for 10 min, as indicated. Cell pellets were collected, lysed in phospho-lysis buffer [50 mM Hepes (pH 7.3), 150 mM NaCl, 1.5 mM MgCl_2_, 1 mM EDTA (pH 8.0), 100 μM sodium fluoride, 10 μM sodium pyrophosphate, 0.5% Triton X-100, and 10% glycerol] supplemented with protease and phosphatase inhibitors, and processed for immunoblotting analyses.

To assess the requirement of PKCδ for IFNα-induced phosphorylation of ULK1 on Ser341 and Ser495, U937 cells were transfected with control (ON-TARGETplus Non-targeting Control Pool, # D-001810-10-05; Dharmacon) or PKCδ (ON-TARGETplus Human PRKCD siRNA SMARTpool, # L-003524-00-0005; Dharmacon) siRNAs using Amaxa Biosystems Nucleofector Kit C and program W-001 (Lonza), as per the manufacturer’s instructions (1 × 10^6^ cells/transfection reaction). The next day, transfected cells were starved overnight (cultured in 0% FBS RPMI medium) and then were either left untreated or were treated with IFNα (10^4^ IU/mL) for 10 or 30 min, as indicated. Cell pellets were collected, lysed in phospho-lysis buffer supplemented with protease and phosphatase inhibitors, and processed for immunoblotting analyses.

For the experiments to evaluate the role of ULK1 phosphorylation on Ser341 and Ser495 on transcription of ISGs, *ULK1* KO KT-1 cells were transfected with myc-tagged EV, ULK1 WT, ULK1 S341A, or ULK1 S495A plasmids using Amaxa Biosystems Nucleofector Kit V and program X-001 (Lonza), as per the manufacturer’s instructions (3 × 10^6^ cells/transfection reaction). Transfection efficiency was assessed by immunoblotting analysis. The next day, cells were either left untreated or were treated with IFNα (5000 IU/mL) for 6 h and total RNA was isolated using the RNeasy Mini Kit (QIAGEN), as per the manufacturer’s instructions.

For the experiments to assess whether ROCK1/2 regulate transcription of ISGs, HEL cells were transfected with either control (ON-TARGETplus Non-targeting Control Pool, # D-001810-10-05; Dharmacon) or ROCK1 (ON-TARGETplus Human ROCK1 siRNA SMARTPool, # L-003536-00-0005; Dharmacon) and ROCK2 (ON-TARGETplus Human ROCK2 siRNA SMARTPool, # L-004610-00-0005; Dharmacon) siRNAs, using Amaxa Biosystems Nucleofector Kit V and program X-005 (Lonza), as per the manufacturer’s instructions (4 × 10^6^ cells/transfection reaction). The next day, cells were either left untreated or were treated with IFNα (1000 IU/mL) for 24 h and total RNA was isolated using the RNeasy Mini Kit (QIAGEN), as per the manufacturer’s instructions.

For the experiments to determine whether IFNα-induced cleavage of ROCK1/2 is caspase-dependent, HEL, U937 and KT-1 cells were either left untreated or treated with IFNα (1000 IU/mL) and/or the caspase inhibitor (CASPi) Z-VAD-FMK (20 μM; Selleckchem Cat No. S7023) for 24 or 48 h, as indicated. Cell pellets were collected, lysed in phospho-lysis buffer supplemented with protease and phosphatase inhibitors, and processed for immunoblotting analyses.

### Co-immunoprecipitation of endogenous protein-ULK1 complexes

To study the physical interaction between ULK1 and PKCδ, HEL, SET-2, KT-1, or U937 cells were either left untreated or were treated with human IFNα (Infergen) or IFNβ (Biogen) (10^4^ IU/mL) for 10 min, as indicated. To study the physical interaction between ULK1 and ROCK1 or ROCK2, HEL cells were either left untreated or were treated with human IFNα (10^4^ IU/mL) for 10 min. After treatment, cell pellets were lysed in NP-40 buffer [20 mM Hepes (pH 7.4), 180 mM KCl, 0.2 mM EGTA, 10% glycerol, and 0.1% NP-40] supplemented with protease and phosphatase inhibitors. For immunoprecipitation (IP) of endogenous protein-ULK1 complexes, 500 μg of protein (total cell lysates) from each sample were incubated overnight at 4 °C with rotation with ULK1 (D8H5) rabbit monoclonal antibody (2.1 μg/mg protein; #8054, Cell Signaling Technology), followed by incubation for 1 h at 4 °C with rotation with protein G Sepharose 4 Fast Flow beads (GE Healthcare). As control, the same procedure was followed using rabbit (DA1E) monoclonal antibody IgG XP Isotype control (2.1 μg/mg protein; #3900, Cell Signaling Technology) instead of ULK1 antibody. After IP, the beads were washed three times with NP-40 buffer without glycerol. Protein-ULK1 complexes were eluted from the beads by incubation with Lane Marker Reducing Sample Buffer (Pierce) at 95 °C for 10 min. Eluates were resolved by SDS-PAGE and processed for immunoblotting analyses.

### Immunoprecipitations and in vitro kinase assays

To determine whether PKCδ could directly phosphorylate ULK1, KT-1 cells were starved overnight and then left untreated or were treated with human IFNβ (10^4^ IU/mL) for 10 and 30 min, as indicated, then lysed in phospho-lysis buffer [50 mM Hepes (pH 7.3), 150 mM NaCl, 1.5 mM MgCl_2_, 1 mM EDTA (pH 8.0), 100 μM sodium fluoride, 10 μM sodium pyrophosphate, 0.5% Triton X-100, and 10% glycerol] supplemented with protease and phosphatase inhibitors. 500 μg of protein (total cell lysates) from each sample were used for IP of PKCδ using PKCδ (D10E2) rabbit monoclonal antibody (0.6 μg/mg protein; #9616, Cell Signaling Technology), followed by incubation with protein G Sepharose 4 Fast Flow beads (GE Healthcare Life Sciences). As a control, the same procedure was followed, but using rabbit monoclonal antibody (DA1E) IgG XP Isotype control (0.6 μg/mg protein; #3900, Cell Signaling Technology), instead of PKCδ antibody. The beads were washed three times with phospho-lysis buffer [50 mM Hepes (pH 7.3), 150 mM NaCl, 1.5 mM MgCl_2_, 1 mM EDTA (pH 8.0), 100 μM sodium fluoride, 10 μM sodium pyrophosphate, 0.1% Triton X-100], and twice with kinase buffer [25 mM Tris-HCl (pH 7.4), 0.5 mM EDTA, 5 mM MgCl_2_, 1 mM DTT, 700 μg/mL of phosphatidylserine), prior to the kinase reaction. In vitro kinase reactions to detect PKCδ kinase activity were performed for 30 min with rotation at room temperature in kinase buffer supplemented with 20 μM of ATP (#V915A; Promega) and 10 μCi of γ-^32^P ATP (#BLU502Z250UC, PerkinElmer Life Science) per reaction. ULK1 recombinant human protein (1.3 μg/reaction; #PV6430, Life Technologies) was heat inactivated at 65 °C for 20 min in a sonicating bath and then used as an exogenous substrate in the kinase reactions. Kinase reactions were stopped by incubation with Lane Marker Reducing Sample Buffer (Pierce) at 95 °C for 10 min. Samples were then resolved by SDS-PAGE, transferred to an Immobilon-P PVDF membrane (Millipore), and processed for autoradiography and subsequent immunoblotting analyses.

To determine the specific PKCδ phosphorylation sites in ULK1, myc-tagged empty vector, ULK1 WT, and S/A ULK1 mutant plasmids were transfected into HEK293T cells and, 24 h later, cell pellets were collected and lysed in NP-40 buffer supplemented with protease and phosphatase inhibitors. For IP of myc-tagged proteins, 5 mg of protein (total cell lysates) from each transfection were incubated with 200 μL of c-Myc antibody (9E10) conjugated to agarose beads (500 μg/mL, 25% agarose; #sc-40 AC; Santa Cruz Biotechnology) for 3 h at 4 °C with rotation. The beads were then washed twice with phospho-lysis buffer, twice with NP-40 buffer without glycerol and twice with kinase dilution buffer III (#K23-09; SignalChem). After the last wash, samples were resuspended in kinase dilution buffer III and 10 μL aliquots were stored at –80 °C. The immunoprecipitated myc-tagged proteins were heat inactivated at 65 °C for 20 min in a sonicating bath and then used as substrates for active recombinant full-length human PKCδ protein (50 ng/reaction; #P64-10G-10; SignalChem) in the presence of γ-^32^P ATP in kinase assays, following the manufacturer’s instructions (SignalChem). Kinase reactions were stopped by incubation with Lane Marker Reducing Sample Buffer (Pierce) at 95 °C for 10 min and samples were then resolved by SDS-PAGE. The gel was stained with Coomassie blue (SimplyBlue SafeStain; Thermo Fisher Scientific) and then dried overnight and processed for autoradiography.

### Generation and validation of phospho-specific ULK1 antibodies

The rabbit polyclonal p-Ser^341^ULK1 antibody was generated against the peptide sequence C-AGFLHS(pS)RDSGGS-amide and the rabbit polyclonal p-Ser^495^ULK1 antibody was generated against the peptide sequence C-VLARKM(pS)LGGGRP-amide and then purified by affinity purification (90% purity peptide) by Pierce/Thermo Fisher Scientific. To confirm the antibodies’ specificity (p-Ser^341^ULK1 antibody used at 1:1000 and p-Ser^495^ULK1 antibody used at 1:500) myc-tagged empty vector, ULK1 WT, and S/A ULK1 mutant plasmids were transfected into *Ulk1/2*^−/−^ MEFs using TurboFect Transfection Reagent (Thermo Fisher Scientific) according to the manufacturer’s protocol and, 24 h later, cells were treated with IFNα (10^4^ IU/mL) for 10 min and then lysed in phospho-lysis buffer supplemented with protease and phosphatase inhibitors, and processed for immunoblotting analyses.

### Immunoblotting analyses

Equal amounts of total-cell lysates were mixed with Lane Marker Reducing Sample Buffer (Pierce), boiled at 95 °C for 5 min, resolved by SDS-PAGE, and then transferred to an Immobilon-P PVDF membrane (Millipore) using the Trans-Blot Turbo transfer system (Bio-Rad). For immunoblotting analyses, the membranes were probed with primary antibodies, followed by horseradish peroxidase–conjugated secondary antibodies, and antibody binding was detected by enhanced chemiluminescence using an Amersham ECL prime Western blotting detection reagent (GE Healthcare Life Sciences). The commercially available antibodies used for western blotting are rabbit monoclonal anti-ULK1 (D8H5; 1:1000; # 8054), rabbit monoclonal anti-PKC-delta (D10E2; 1:1000; # 9616), rabbit polyclonal anti-GST (1:1000; # 2622), rabbit monoclonal anti-Phospho-p38 MAPK (Thr180/Tyr182) (3D7; 1:1000; # 9215), rabbit monoclonal anti-p38 MAPK (D13E1) XP (1:1000; # 8690), rabbit monoclonal anti-ROCK1(C8F7) (1:1000; # 4035), rabbit monoclonal anti-ROCK2 (D1B1) (1:1000; # 9029), rabbit monoclonal anti-phospho-ULK1 (Ser757) (D7O6U) (1:1000; # 14202), rabbit polyclonal anti-caspase 3 (1:1000; # 9662), and rabbit polyclonal anti-PARP (1:1000; # 9542) from Cell Signaling Technology, rabbit polyclonal anti-PKC Delta (1:1000, # 14188-1-AP) from Proteintech and the mouse monoclonal anti-GAPDH clone 6C5 (1:20,000; # MAB374) from EMD Millipore. Bands corresponding to proteins of interest were scanned and quantified by densitometry using ImageJ software.

### Mass spectrometry

To identify proteins that interact with endogenous ULK1, HEL cells were either left untreated or treated with human IFNα (10^4^ IU/mL) for 10 min, then lysed in NP-40 buffer [20 mM Hepes (pH 7.4), 180 mM KCl, 0.2 mM EGTA, and 0.1% NP-40] supplemented with protease and phosphatase inhibitors. Three milligrams of protein (total cell lysates) from untreated and IFNα-treated samples was used for immunoprecipitation of endogenous protein-ULK1 complexes using ULK1 (D8H5) rabbit monoclonal antibody. As control, the same procedure was followed for IFNα-treated lysates, but using rabbit (DA1E) monoclonal antibody immunoglobulin G (IgG) XP isotype control instead of the ULK1 antibody. After incubation overnight with rotation at 4 °C, samples were incubated for 1 h at 4 °C with rotation with protein G Sepharose 4 Fast Flow beads (50 μL/sample; #17-0618-01; GE Healthcare Life Sciences) and then washed three times with NP-40 buffer and twice with wash buffer [20 mM Hepes (pH 7.4), 180 mM KCl, and 0.2 mM EGTA]. Protein-ULK1 complexes were eluted from the beads by incubation with Lane Marker Reducing Sample Buffer (Pierce) at 95 °C for 10 min, and proteomic analyses were performed in the Northwestern Proteomics Core Facility (Northwestern University, Chicago). IP-eluted proteins were initially separated using SDS-PAGE and the gel then cut into 10 equivalent height bands before standard in-gel digestion^70^. Resulting peptides were extracted from the gel pieces and desalted using solid-phase extraction on a Pierce C18 Spin column, before elution in 40 μL of 80% acetonitrile in 0.2% formic acid. After lyophilization, peptides were reconstituted with 0.1% formic acid in water and injected onto a trap column (150 μm; inner diameter [ID] by 3 cm) coupled with a nanobore analytical column (75 μm; ID by 15 cm; both ReproSil-Pur C18-aQ, 3 μm. Samples were separated using a linear gradient of solvent A (95% water, 5% acetonitrile, and 0.1% formic acid) and solvent B (5% water, 95% acetonitrile, and 0.1% formic acid) over 60 min. nLC-MS/MS data were obtained on a Velos Orbitrap (Thermo Fisher Scientific) mass spectrometer. Data were searched using Mascot 2.5 (Matrix Science, http://www.matrixscience.com/index.html) against the human SwissProt database (https://www.uniprot.org/uniprot/?query=reviewed:yes) to identify the proteins processed by nLC-MS/MS, and results were reported at 1% false-discovery rate in Scaffold 4 (Proteome Software). Proteins identified by nLC-MS/MS analysis in the control group (rabbit IgG) were excluded from our data analysis.

### DepMap data analysis

Proteomic data for ROCK1 (Q13464) and ROCK2 (O75116) proteins were downloaded from the public DepMap portal (https://depmap.org/portal/) for a total of 375 cell lines and the data were graphed according to primary disease.

### Cellular viability assays

In the experiments to assess the effects of silencing ROCK1/2 on IFNα-induced anti-proliferative responses, SET-2 cells were transfected with either control (ON-TARGETplus Non-targeting Control Pool, # D-001810-10-05; Dharmacon) or ROCK1 (ON-TARGETplus Human ROCK1 siRNA SMARTPool, # L-003536-00-0005; Dharmacon) and ROCK2 (ON-TARGETplus Human ROCK2 siRNA SMARTPool, # L-004610-00-0005; Dharmacon) siRNAs using Amaxa Biosystems Nucleofector Kit V and program X-013 (Lonza) as per the manufacturer’s instructions. 24 h later, control siRNA or ROCK1/2 siRNA-transfected cells (knockdown efficiency was confirmed by immunoblotting analyses) were seeded (10,000 cells/well) in quadruplicate in individual wells of 96-well plates in the absence or presence of human IFNα (100  IU/mL), for 5 days. In the experiments to assess the effects of drug-targeted inhibition of ROCK1/2 on IFNα-induced anti-proliferative responses, SET-2 cells were seeded (10,000 cells/well) in quadruplicate in wells of 96-well plates and treated with vehicle-control (DMSO), GSK429286A (20 μM; #73182; StemCell Technologies), and/or human IFNα (100 IU/mL) for 5 days. In parallel experiments, HEL cells were seeded (1500 cells/well) in quadruplicate in wells of 96-well plates and treated with vehicle-control (DMSO), GSK429286A (30 μM) or Fasudil (30 μM; #73662; StemCell Technologies) and/or human IFNα (1000 IU/mL) for 5 days. Cell viability and proliferation were quantified using Cell Proliferation Reagent WST-1 (Hoffman-La Roche Inc./Sigma), according to the manufacturer’s protocol and the absorbance was measured using the Epoch microplate spectrophotometer and Gen5 software (BioTek).

### Hematopoietic cell progenitor assays

In the experiments to assess the role of PKCδ or ROCK1/2 on IFNα-mediated anti-clonogenic effects, primary PBMCs from PV patients were transfected with control or PKCδ (ON-TARGETplus Human PRKCD siRNA SMARTPool, # L-003524-00-0005, Dharmacon), or ROCK1 and ROCK2 siRNAs using *Trans*IT-TKO Transfection Reagent (Mirus), as per the manufacturer’s instructions, as indicated. Hematopoietic progenitor colony formation for human erythroid precursors (burst-forming unit erythroid [BFU-E]) was then determined in clonogenic assays in MethoCult H4434 Classic medium (Stemcell Technologies) in the absence or presence of human IFNα (1000 IU/mL). In the experiments to assess the effects of drug-targeted inhibition of ROCK1/2 on IFNα-mediated anti-clonogenic effects, HEL cells were seeded in MethoCult H4534 Classic medium (Stemcell Technologies) and treated with vehicle-control (DMSO), GSK429286A (5 μM), and/or human IFNα (100 IU/mL). In parallel, primary PBMCs from PV patients were seeded in MethoCult H4434 Classic medium (Stemcell Technologies) and treated with vehicle-control (PBS), Fasudil (30 μM), and/or human IFNα (1000 IU/mL). Hematopoietic colony formations were scored as in previous studies^21,71^. Percent (%) of colony formation was calculated by dividing each colony count by the average colony count in the control group, and multiplying by 100%.

### Quantitative RT-PCR

Total RNA was isolated from bone marrow or PBMCs banked at the New York Blood Center and that were harvested before starting PEG-IFNα treatment from MPN patients enrolled in the Myeloproliferative Disorders Research Consortium (MPD-RC)-111 study (clinical trial #NCT01259817)^7^ using the RNeasy Mini Kit (QIAGEN) as per the manufacturer’s instructions. Two micrograms of total cellular mRNA were reverse-transcribed into cDNA using the Omniscript RT kit (QIAGEN) and oligo(dT)12–18 primers (Life Technologies). Quantitative RT-PCR was carried out using a Bio-Rad CFX96 Real Time System (Bio-Rad), using commercially available fluorescein amidite (FAM)–labeled primer/probe sets (Thermo Fisher Scientific) to determine human PRKCD (PKCδ) (Hs00178914_m1), ULK1 (Hs00177504_m1) and MAPK14 (p38 MAPK) (Hs01051152_m1) mRNA expression. Human 18S (Hs99999901_s1) expression was used as a reference gene and to calculate delta Ct (ΔCt) for each gene in each patient. Results were then matched to response status to PEG-IFNα therapy for each patient and statistical analyses were performed as indicated in the respective figure legend.

For the qRT-PCR studies using transfected *ULK1* KO KT-1 cells and transfected HEL cells, two micrograms of total cellular mRNA were reverse-transcribed into cDNA using the High Capacity cDNA Reverse Transcription Kit (Applied Biosystems). Quantitative RT-PCR was carried out using a Bio-Rad CFX96 Real Time System (Bio-Rad) using commercially available fluorescein amidite (FAM)–labeled primer/probe sets (Thermo Fisher Scientific) to determine human ROCK1 (Hs01127701_m1), ROCK2 (Hs00178154_m1), IFIT1 (Hs00356631_g1), OAS1 (Hs00973635_m1), IFIT3 (Hs01922752_s1), IRF7 (Hs01014809_g1) and IFI6 (Hs00242571_m1). Human GAPDH (Hs03929097_g1) expression was used for normalization and to calculate delta Ct (ΔCt) for each gene for each experimental condition. The mRNA expression was calculated using the ΔΔCt method, and the data were plotted as the increase in fold change compared with untreated samples. Ct values were collected using the Bio-Rad CFX Maestro software.

### Gene expression analyses in MPN patients

Levels of *ROCK1* (ID: 213044_at) and *ROCK2* (ID: 211504_x_at) mRNA expression in peripheral blood neutrophils isolated from healthy individuals and patients with ET, MF, and PV were extracted from the GSE54646 dataset^72^ accessible at NCBI GEO database, and Log2 of gene expression was calculated for each gene.

### Mice bone marrow transplantation and treatment

*Jak2*^V617F^ conditional knock-in (KI) mice (CD45.2)^30^ were provided by B. L. Ebert and A. Mullally (Harvard Medical School). Vav-Cre^+^ (CD45.2) (B6.Cg-Commd10^Tg(Vav1-icre)A2Kio^/J) (Stock No: 008610) and wild-type CD45.1 (B6.SJL-*Ptprc*^a^*Pepc*^b^/BoyJ) (Stock No. 002014) mice were obtained from Jackson Laboratory. *Jak2*^V617F/+^ KI mice were crossed with Vav-Cre^+^ mice to produce *Jak2*^V617F/+^VavCre+ and *Jak2*^+/+^VavCre- female and male donor mice. *Jak2*^V617F/+^VavCre^+^ mice express *Jak2*^V617F^ under the control of the hematopoietic-specific Vav promoter^73^. For genotyping, tail-tips were collected at weaning, digested, then recovered DNA was amplified using the following primers: Vav-Cre F: 5’-AGA TGC CAG GAC ATC AGG AAC CTG-3’, Vav-Cre R: 5’-ATC AGC CAC ACC AGA CAC AGA GAT C-3’ and JAK2-F: 5’-CGT GCA TAG TGT CTG TGG AAG TC-3’, JAK2-R: 5’-CGT GGA GAG TCT GTA AGG CTC AA-3’. CD45.1 recipient female and male mice were subjected to 11 gray (Gy) units of ionizing radiation. One week prior to and 2-weeks following irradiation, mice were fed bactrim-supplemented water. Two to 3 h after irradiation, mice were injected intravenously with 2 × 10^6^ bone marrow *Jak2*^V617F/+^VavCre+ or *Jak2*^+/+^VavCre- cells, post red blood cell lysis, into the tail vein or retro-orbitally. Peripheral blood (PB) was collected and hematocrit, hemoglobin levels, and chimerism (engraftment) were measured 3 or 5 weeks after transplantation. Mice were randomized into treatment groups by hematocrit and/or hemoglobin levels. To test the effects of IFNα treatment on ROCK1/2 activation, recipient mice (Fig. 5a [10–11-week-old female mice vehicle-control group: *n* = 5 and IFNα groupː *n* = 6] and Fig. 6a [10–11-week-old female mice vehicle-control group: *n* = 5 and IFNα groupː *n* = 5, study was repeated with 10–11-week-old male mice vehicle-control group: *n* = 5 and IFNα groupː *n* = 5]) or C57BL/6J mice (Supplementary Fig. 8a [9–10-week-old female mice vehicle-control group: *n* = 10 and IFNα groupː *n* = 10]) were treated once a week for 4 weeks with murine ropeginterferon-α (PEG-IFN-α; PharmaEssentia; 600 ng in 500 μL PBS, subcutaneous injection) or with vehicle (PBS). To test the effects of drug-targeted inhibition of ROCK1/2 in combination with IFNα treatment in vivo (Fig. 8a), recipient mice were treated for 4 weeks with Fasudil (FAS; # 10010559; Cayman Chemical; 25 mg/kg, intraperitoneal injection) four times per week or vehicle (PBS), and/or with PEG-IFNα (600 ng in 500 μL PBS, subcutaneous injection) once a week or vehicle (PBS), and analyzed 3 days after the last injection. We used 3–6 mice (10–11 week old) per treatment group as the number of recipient mice that we are able to transplant is dependent on the number of the same sex and age of *Jak2*^VF/+^VavCre^+^ donor mice that we can generate by breeding. We combined the data from four independent studies in the same plot for the different parameters studied (Fig. 8 and Supplementary Figs. 10 and 11 CTRL: *n* = 8 females and *n* = 7 males; FAS: *n* = 10 females and *n* = 7 males; IFNα: *n* = 8 females and *n* = 7 males; FAS + IFNα: *n* = 10 females and *n* = 8 males).

### Blood count analysis and isolation of BM cells for immunoblotting

Peripheral blood (PB) was collected in EDTA-coated tubes (VWR) and hematocrit, hemoglobin, red blood cells and platelets levels were analyzed on the Hemavet (Model 950, Drew scientific) and data collected using the Hemavet 950 software. BM cells were flushed, homogenized through a 70 µm cell strainer (Fisher Scientific/Falcon, Cat # 352350) and red blood cells (RBCs) were lysed with 1x RBC lysis buffer (eBioscience 10x RBC Lysis Buffer, Thermo Fisher Scientific/invitrogen, Cat # 00-4300-54) prior to total protein isolation, using phospho-lysis buffer for immunoblotting analyses.

### Flow cytometry analysis

To determine the efficiency of engraftment of CD45.2+ bone marrow cells, PB was collected in EDTA-coated tubes, red blood cells were lysed using 1x RBC lysis buffer, then cells were stained in FACS buffer (PBS, 0.5% BSA, 1 mM EDTA) using the antibodies: PerCP-Cy5.5 mouse anti-mouse CD45.2 (Clone 104; BD Pharmigen) (1:200; # 561096) and V450 mouse anti-mouse CD45.1 (Clone A20; BD Horizon) (1:200; # 560520) from BD Biosciences. Unstained and single color controls were used for each experiment. Percentage of engraftment was defined as the proportion of Jak2^+/VF^ cells as a percentage of total mononuclear cells in peripheral blood. Calculation: (%Jak2^+/VF^)/(%Jak2^+/VF^  +  %WT) × 100. Population frequencies were calculated as a percentage of the respective donor CD45 expressing cells (WT-CD45.1; Jak2^+/VF^-CD45.2).

To assess the effects of PEG-IFNα and/or Fasudil treatments on phosphorylation of MYPT1 in PBMCs, PB was collected in EDTA-coated tubes and red blood cells were lysed using 1x RBC lysis buffer. PBMCs were then fixed, permeabilized and stained using the BD Cytofix/Cytoperm Fixation/Permeabilization Solution Kit (# BD 554714) and the mouse monoclonal anti-p-MYPT1 (F-11) Alexa Fluor 647 (1:50; Santa Cruz Biotechnology # sc-377531 AF647) or normal mouse IgG1 Alexa Fluor 647 (negative control, 1:50; Santa Cruz Biotechnology # sc-24636) antibodies, according to the manufacturer’s instructions. Unstained and negative controls were used for each experiment.

To assess the effects of PEG-IFNα treatment on phosphorylation of MYPT1 in BMMCs, BM cells were flushed, homogenized through a 70 µm cell strainer, and red blood cells were lysed using 1x RBC lysis buffer. BMMCs were then fixed, permeabilized and stained using the BD Cytofix/Cytoperm Fixation/Permeabilization Solution Kit (# BD 554714) and the PE/Cyanine7 anti-mouse CD71 (1:200; BioLegend # 113812), the mouse monoclonal anti-p-MYPT1 (F-11) Alexa Fluor 647 (1:50; Santa Cruz Biotechnology # sc-377531 AF647) or the normal mouse IgG1 Alexa Fluor 647 (negative control, 1:50; Santa Cruz Biotechnology # sc-24636) antibodies, according to the manufacturer’s instructions. Unstained, single color and negative controls were used for each experiment.

To assess the effects of PEG-IFNα and/or Fasudil treatments on erythroid development, BM cells were flushed and homogenized through a 70 µm cell strainer, and spleens were harvested, weighed and then mechanically dissociated and homogenized through a 70 µm cell strainer. BM and spleen cells were then stained in FACS buffer using the antibodies: APC anti-mouse TER-119/Erythroid Cells (1:200; BioLegend # 116212) and PE/Cyanine7 anti-mouse CD71 (1:200; BioLegend # 113812). Unstained and single color controls were used for each experiment.

To assess the effects of PEG-IFNα and/or Fasudil treatments on myeloid progenitor cells, BM cells were flushed and homogenized through a 70 µm cell strainer, red blood cells were lysed with 1x RBC lysis buffer and BM mononuclear cells were then stained in FACS buffer using V450 Mouse Lineage Antibody Cocktail (1:20; BD Biosciences # 561301), Ly-6A/E (Sca-1) Monoclonal Antibody (D7), PE-Cyanine7, eBioscience (1:100; Invitrogen/Thermo Fisher Scientific # 25-5981-82), APC Rat Anti-Mouse CD117 (c-kit) antibody (1:100; BD Biosciences # 553356), PE Rat anti-Mouse CD34 antibody (1:40; BD Biosciences # 551387), and PerCP-Cy5.5 Rat Anti-Mouse CD16/CD32 antibody (1:200; BD Biosciences # 560540). Unstained and single color controls were used for each experiment.

All samples were analyzed by flow cytometry using a BD LSRII flow cytometer, the data acquired using BD FACSDiva, and analyzed using FlowJo software v10.6 (Treestar, CA).

### Quantification and statistical analysis

All statistical analyses were performed with GraphPad Prism 8, except for qRT-PCR and colony formation assays using PV patient-derived samples for which R package lme4 version 1.1.26 (https://cran.r-project.org/web/packages/lme4/index.html) was used^74,75^. Unless otherwise indicated in the figure legend, plots shown are representative of at least three independent biological replicates or from samples collected from at least three different patients or three different mice. Sample size for each experimental group/condition is reported in the respective figure legend. For mouse experiments, each data point represents data for one individual mouse. Statistically significant differences were determined using two-sample two-tailed *t*-test when comparing a normally distributed outcome between two groups. When comparing more than two groups, significance was determined using one-way ANOVA with Tukey’s post-hoc test for multiple comparisons correction or with Dunnett’s for multiple comparisons to a single control group. Two-way ANOVA was used to compare groups in experiments with two independent variables (factors) with Tukey’s post-hoc test for multiple comparisons correction. For the experiments using MPN-patient-derived primary cells, differences between experimental conditions were analyzed using linear mixed effects models with patient as a random effect to account for the within-subject correlation between multiple conditions. Kenward-Roger degrees of freedom adjustment was used, and pairwise group comparison tests were adjusted for multiple comparisons using Tukey’s method. The statistical analysis performed for each dataset is included in the figure legends. Differences were considered statistically significant when *p*-values were less than 0.05.

### Reporting summary

Further information on research design is available in the Nature Research Reporting Summary linked to this article.

## Supplementary information

Supplementary Information

Reporting summary

## Data Availability

The mass spectrometry proteomics data generated in this study have been deposited to the ProteomeXchange Consortium via the PRIDE^76^ partner repository with the dataset identifier PXD021748 and 10.6019/PXD021748. The ROCK1/2 gene expression data in MPN patients vs. healthy individuals used in this study are available in the NCBI GEO database under accession code GSE54646 and within the Source Data file of this manuscript. The proteomic data for ROCK1 (Q13464) [https://depmap.org/portal/gene/ROCK1?tab=characterization&characterization=proteomics] and ROCK2 (O75116) [https://depmap.org/portal/gene/ROCK2?tab=characterization&characterization=proteomics] proteins are available in the public DepMap portal and within the Source Data file of this manuscript. The authors declare that all other data that support the findings of this study are available within the paper, its supplementary information, or Source Data file. Source data are provided with this paper.

## Source data

Source Data
