# Supplementary material for: Discovery of a signaling feedback circuit that defines interferon responses in myeloproliferative neoplasms
Source: Nat Commun. 2022 Apr 1;13:1750. doi: 10.1038/s41467-022-29381-7 (PMC8975834; doi:10.1038/s41467-022-29381-7)
Supplement: Supplementary file 1 — Supplementary Information [file 41467_2022_29381_MOESM1_ESM.pdf]

Discovery of a Signaling Feedback Circuit that Defines Interferon Responses in Myeloproliferative Neoplasms

Supplementary information

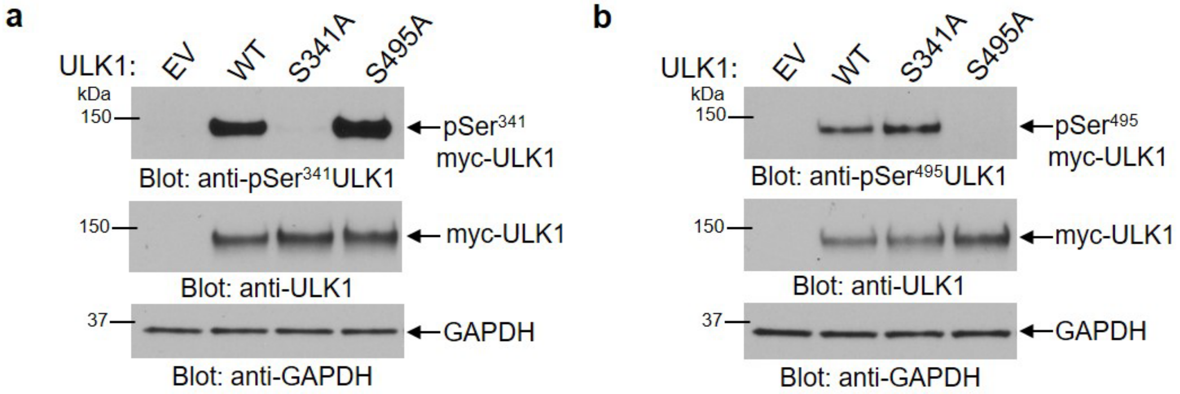

Supplementary Fig. 1. Specificity of phospho-Ser^{341}-ULK1 and phospho-Ser^{495}-ULK1 antibodies. Related to Fig. 2a.

a-b. Immunoblotting analysis of pSer^{341}-ULK1 and pSer^{495}-ULK1 in lysates from *Ulk1/2*$^{-/-}$ MEFs transfected with myc-tagged empty vector (EV), ULK1 WT, ULK1^{S341A} (S341A) or ULK1^{S495A} (S495A) plasmids and cultured in DMEM medium supplemented with 10% FBS overnight, followed by treatment with IFNα (10^4 IU/mL) for 10 minutes. Blots are representative of three independent experiments. Source data are provided as a Source Data file.

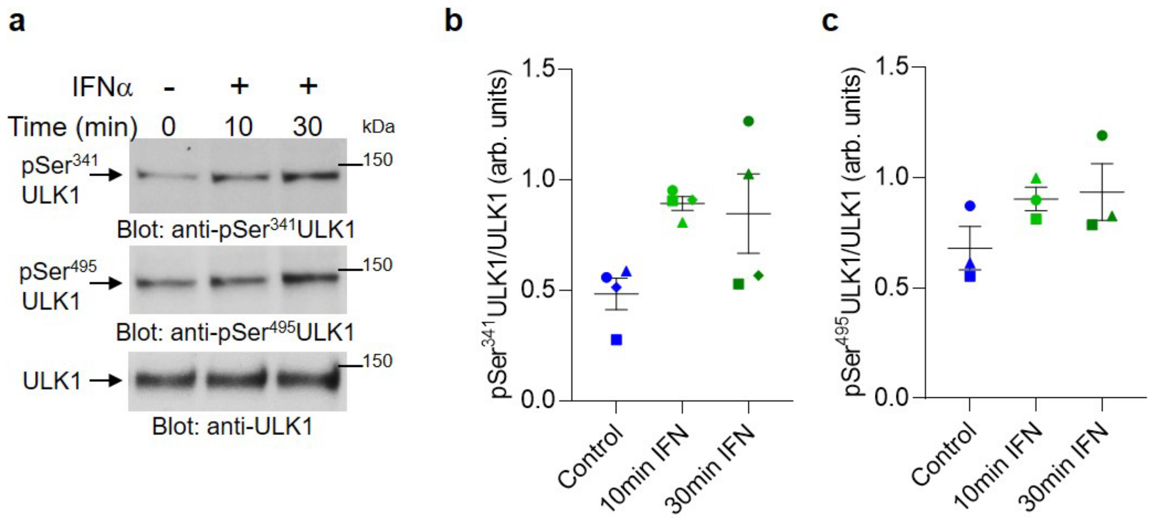

Supplementary Fig. 2. Type I IFN-induces phosphorylation of ULK1 on serine residues 341 and 495. Related to Fig. 2a.

a. Immunoblotting analysis of pSer^{341}-ULK1 and pSer^{495}-ULK1 in lysates from *Ulk1/2*$^{+/+}$ MEFs starved overnight (cultured in DMEM without FBS) followed by treatment with IFNα (10^4 IU/mL) for 10 and 30 minutes, as indicated. Blots are representative of four independent experiments for pSer^{341}-ULK1 and three independent experiments for pSer^{495}-ULK1. The samples derive from the same experiment and the gels/blots were processed in parallel as shown in the source data provided as a Source Data file. GAPDH (loading control) was performed for all the gels/blots and can be seen in the source data provided at the end of this document. **b-c**. Bands for the indicated proteins were scanned and quantified by densitometry using ImageJ software. Quantified data are means ± SEM of p-ULK1/ULK1 for each experimental condition from (b) four and (c) three independent experiments. Each independent experiment is represented by the same symbol. arb.units, arbitrary units. Source data are provided as a Source Data file.

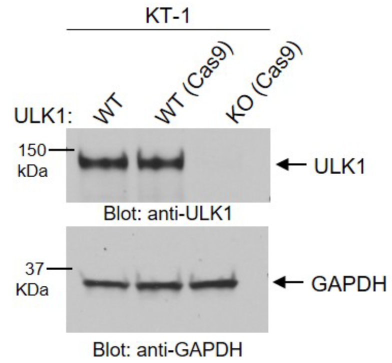

Supplementary Fig. 3. Confirmation of genetic deletion of *ULK1* in KT-1 cells using CRISPR-Cas9 genome editing. Related to Fig. 2e.

Western blot analysis of ULK1 in lysates from wild-type (WT) unmodified KT-1 cells, Cas9-expressing WT KT-1 cells (WT (Cas9)) and Cas9-expressing *ULK1* KO KT-1 (KO (Cas9)) cells generated by CRISPR-Cas9 genome editing. Blots are representative of three independent experiments. Source data are provided as a Source Data file.

Supplementary Table 1. Information on the MPN patients (9 males, 9 females, age range 36-84 years, median age 67) enrolled in clinical trial #NCT01259817, for which the baseline mRNA expression levels of *PRKCD* (PKCδ), *ULK1* and *MAPK14* (p38 MAPK) were considered in relation to clinical response to pegylated-rIFN-α2a treatment (Pegasys). Related to Figure 3b.

Patient No.	Diagnosis	Mutation	Gender	Response
1	PV	JAK2	Female	NR
2	PV	JAK2	Female	R
3	PV	JAK2	Female	NR
4	PV	JAK2	Female	R
5	PV	JAK2	Female	NR
6	ET	CALR	Male	R
7	PV	JAK2	Male	R
8	PV	JAK2	Male	NR
9	PV	JAK2	Female	NR
10	ET	CALR	Male	R
11	PV	JAK2	Male	NR
12	PV	JAK2	Female	R
13	ET	N/A	Female	NR
14	PV	JAK2	Male	R
15	PV	JAK2	Male	R
16	PV	JAK2	Female	R
17	PV	JAK2	Male	NR
18*	PV	JAK2	Male	NR

NR – nonresponse; R - response

*Patient had initially a partial response at 12 months, but presented non-response at 24 months.

N/A – not available

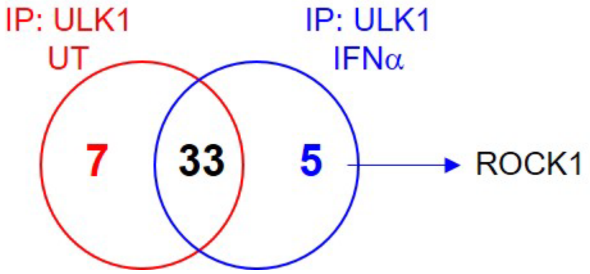

Supplementary Fig. 4. nLC-MS/MS analysis of protein-ULK1 complexes from untreated (UT) or IFNα–treated *JAK2*^{V617F} HEL cells. Related to Fig. 4.

Venn diagram indicates the number of putative proteins that interact with endogenous ULK1 under untreated (red), both (black), and after 10 minutes of IFNα treatment (blue) conditions. ROCK1 was identified as a putative binding partner of ULK1 upon IFNα treatment. The mass spectrometry proteomics data have been deposited to the ProteomeXchange Consortium via the PRIDE[1] partner repository with the dataset identifier PXD021748 and 10.6019/PXD021748.

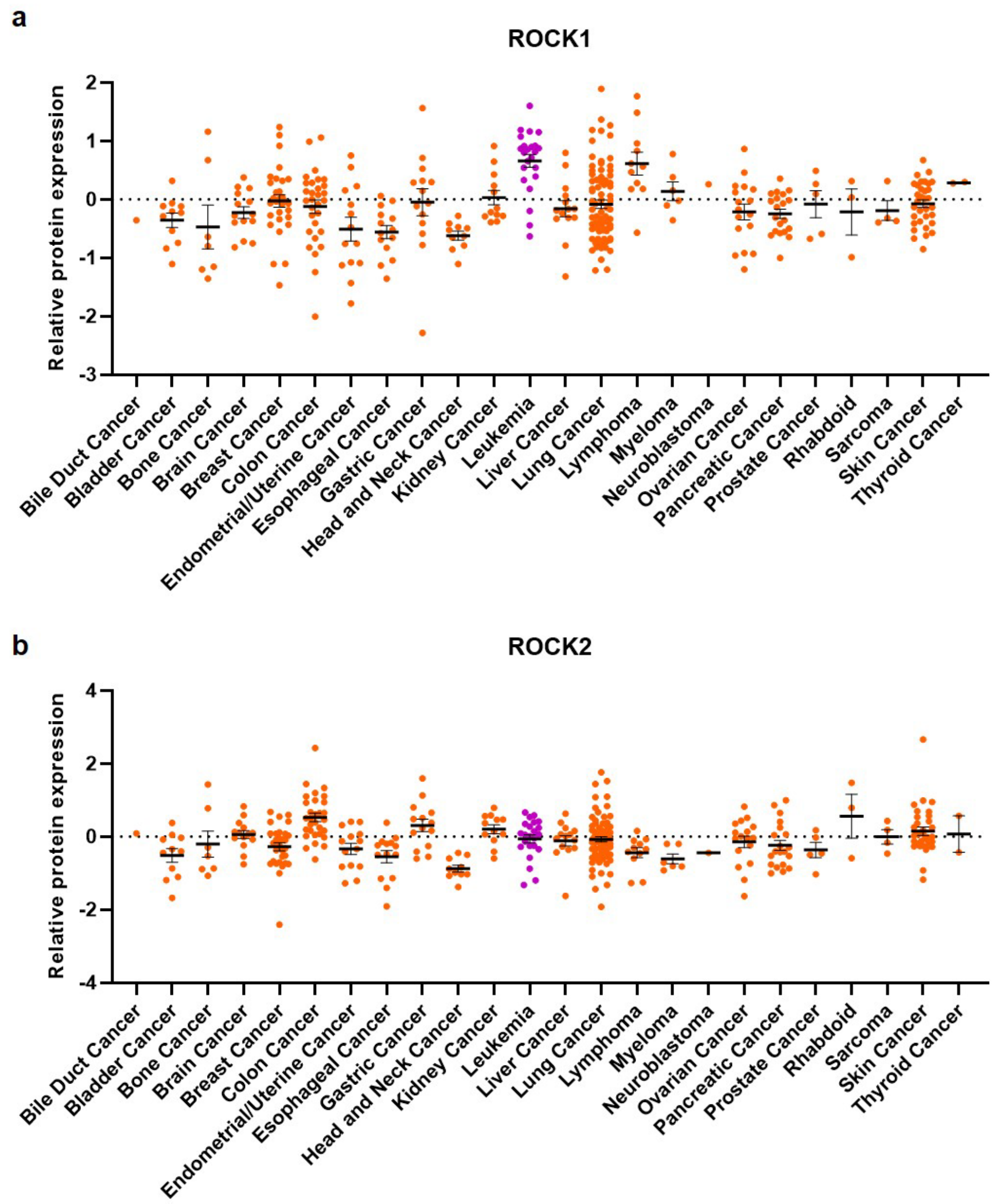

Supplementary Fig. 5. ROCK1/2 protein expression in different types of cancer. Related to Fig. 4.

a-b. Relative protein expression of (**a**) ROCK1 (Proteomics Q13464 dataset) and (**b**) ROCK2 (Proteomics O75116 dataset) in leukemia cell lines (pink) versus other types of cancer cell lines (orange), as indicated. Data shown are means ± SEM of relative protein expression extracted from DepMap Portal (https://depmap.org/portal/). Each data point represents a different cell line (n = 375). Source data are provided as a Source Data file.

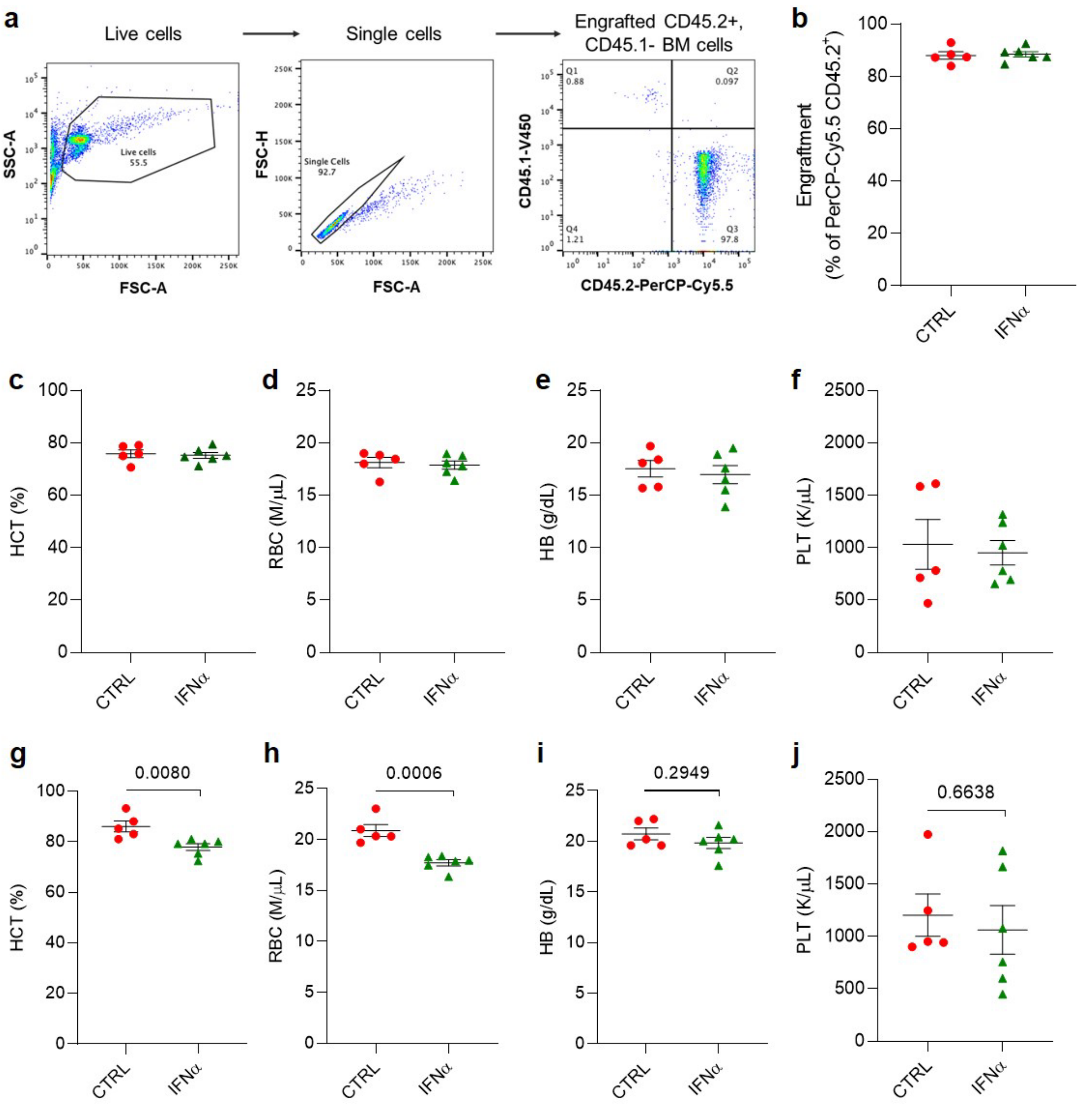

Supplementary Fig. 6. Engraftment, hematocrit, red blood cells, hemoglobin and platelet counts prior to and post PEG-IFNα treatment of *Jak2*$^{V617F/+}$ knock-in mice. Related to Fig. 5. **a-f.** CD45.1 mice were transplanted with *Jak2*$^{V617F/+}$ KI bone marrow cells isolated from *Jak2*$^{V617F/+}$VavCre+ CD45.2 donor mice. Five weeks later, peripheral blood was collected and engraftment, hematocrit (HCT), red blood cells (RBC), hemoglobin (HB) and platelet (PLT) levels

were analyzed and recipient mice were randomized into treatment groups. **a.** Representative image of flow cytometry gating to determine the percentage of engraftment of CD45.2+ cells shown in Figure S6b using CD45.1 and CD45.2 antibodies. Same gating approach was applied for results shown in Figures S7a and S10a. **b-f.** Scatter dot plots show (**b**) engraftment (% of PerCP-Cy5.5 $CD45.2^+$ cells in peripheral blood), (**c**) hematocrit (HCT), (**d**) red blood cells (RBC), (**e**) hemoglobin (HB) and (**f**) platelet (PLT) counts of recipients from vehicle-control (CTRL, n = 5) and PEG-IFNα (IFNα, n = 6) groups, before initiation of treatment. Data shown are means ± SEM. Each data point represents an individual mouse. **g-j.** Five weeks after transplantation and confirmation of engraftment, CD45.1 recipient mice were treated with murine PEG-IFNα once a week (600 ng/mouse, subcutaneous injection) for four weeks or vehicle (PBS). Four weeks after initiation of PEG-IFNα treatment, peripheral blood was collected from vehicle-treated (CTRL, n = 5) and PEG-IFNα-treated mice (IFNα, n = 6). Scatter dot plots show (**g**) hematocrit (HCT), (**h**) red blood cells (RBC), (**i**) hemoglobin (HB) and (**j**) platelet (PLT) counts for each treatment group. Each data point represents an individual mouse. Statistical analyses were performed using two-sample two-tailed t-test. *p* values are reported. Source data are provided as a Source Data file.

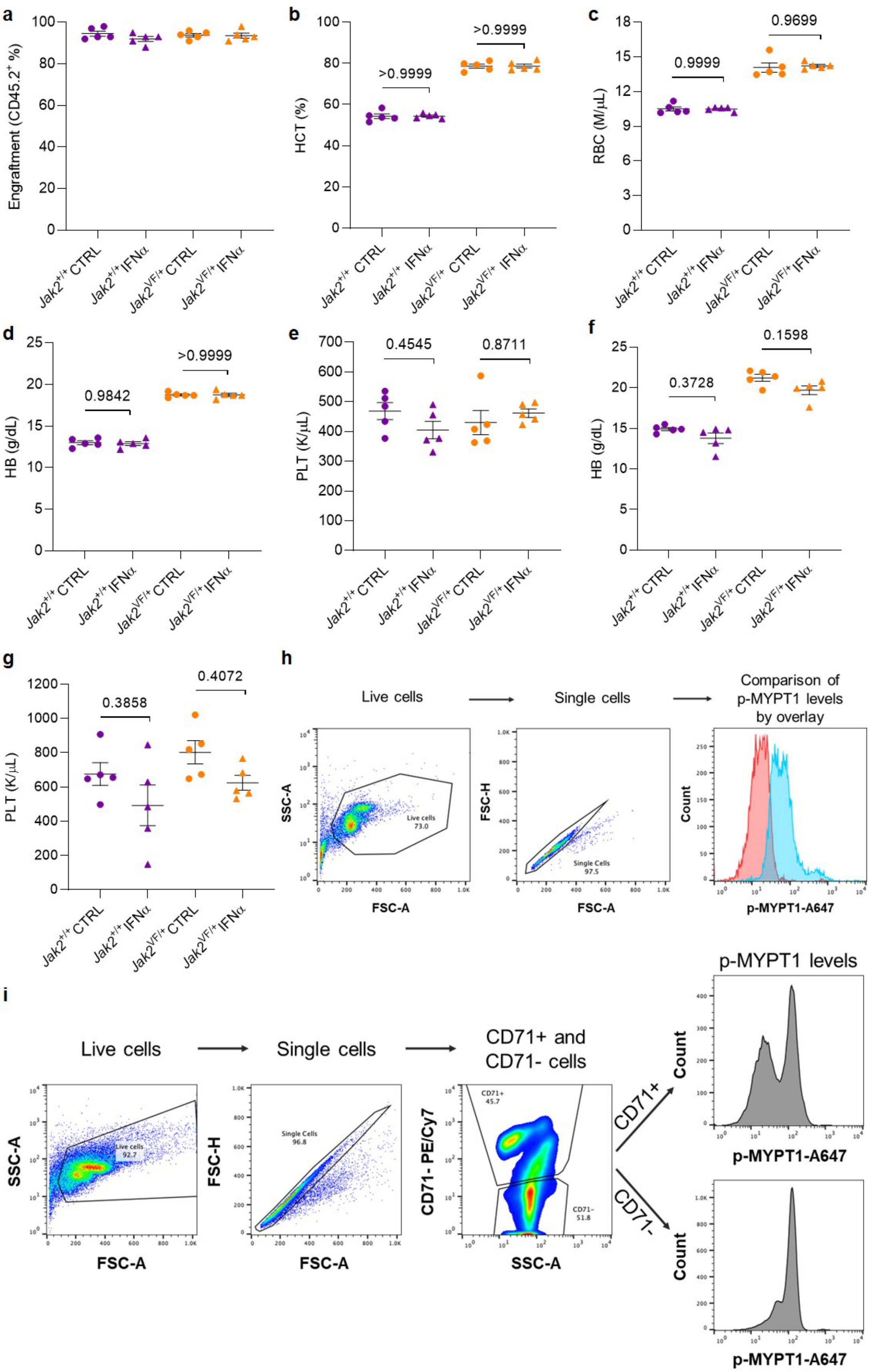
a
Engraftment (CD45.2+ %)
b
HCT (%)
>0.9999
>0.9999
c
RBC (M/µL)
0.9999
0.9699
d
HB (g/dL)
0.9842
>0.9999
e
PLT (K/µL)
0.4545
0.8711
f
HB (g/dL)
0.3728
0.1598
g
PLT (K/µL)
0.3858
0.4072
Jak2+/+ CTRL
Jak2+/+ IFNα
Jak2VF/+ CTRL
Jak2VF/+ IFNα
h
Live cells
Single cells
Comparison of p-MYPT1 levels by overlay
SSC-A
FSC-A
Live cells 73.0
FSC-H
Single Cells 97.5
Count
p-MYPT1-A647
i
Live cells
Single cells
CD71+ and CD71- cells
p-MYPT1 levels
Live cells 92.7
Single Cells 96.8
CD71+ 45.7
CD71- 51.8
CD71- PE/Cy7
SSC-A
FSC-A
FSC-H
CD71+
CD71-
Count
p-MYPT1-A647

Supplementary Fig. 7. Engraftment, hematocrit, red blood cells, hemoglobin and platelet counts prior to and post PEG-IFNα treatment of *Jak2*$^{+/+}$ and *Jak2*$^{V617F/+}$ knock-in recipient mice. Related to Fig. 6.

a-e. CD45.1 mice were transplanted with *Jak2*$^{+/+}$ (in purple) or *Jak2*$^{V617F/+}$ KI (in orange) bone marrow cells isolated from *Jak2*$^{+/+}$VavCre- CD45.2 or *Jak2*$^{V617F/+}$VavCre+ CD45.2 donor mice, respectively. Three weeks later, peripheral blood was collected and engraftment, hematocrit, red blood cells, hemoglobin and platelet levels were analyzed and recipient mice were then randomized into treatment groups. Scatter dot plots show (**a**) engraftment (% of PerCP-Cy5.5 CD45.2^{+} cells in peripheral blood, the gating strategy is shown in Figure S6a), (**b**) hematocrit (HCT), (**c**) red blood cells (RBC), (**d**) hemoglobin (HB) and (**e**) platelet (PLT) counts of recipients from vehicle-control (circles: *Jak2*$^{+/+}$ CTRL n = 5 and *Jak2*$^{VF/+}$ CTRL n = 5) and PEG-IFNα (triangles: *Jak2*$^{+/+}$ IFNα n = 5 and *Jak2*$^{VF/+}$ IFNα n = 5) groups before initiation of treatment. Data shown are means ± SEM. Each data point represents an individual mouse. **f-g.** Three weeks after transplantation and confirmation of engraftment, CD45.1 recipient mice were treated with murine PEG-IFNα once a week (600 ng/mouse, subcutaneous injection) for four weeks or vehicle (PBS). Peripheral blood was collected after the last treatment from vehicle-treated (circles: *Jak2*$^{+/+}$ CTRL n = 5 and *Jak2*$^{VF/+}$ CTRL n = 5) and PEG-IFNα-treated mice (triangles: *Jak2*$^{+/+}$ IFNα n = 5 and *Jak2*$^{VF/+}$ IFNα n = 5). Scatter dot plots show (**f**) hemoglobin (HB) and (**g**) platelet (PLT) counts for each treatment group. Each data point represents an individual mouse. **b-g.** Statistical analyses were performed using two-way ANOVA with treatment (CTRL vs. IFNα), mutation (*Jak2*$^{+/+}$ vs. *Jak2*$^{VF/+}$) and their interaction as predictors. (b-d and f) Regardless of treatment, *Jak2*$^{+/+}$ recipient mice show lower HCT, RBC and HB counts than *Jak2*$^{VF/+}$ recipient mice ($p < 0.0001$). (e and g) The difference in PLT counts between *Jak2*$^{+/+}$ and *Jak2*$^{VF/+}$ recipient mice is not statistically significant ($p > 0.05$). Model-based pairwise comparisons were performed, and *p*-values were adjusted for multiple comparisons using Tukey's method. Adjusted *p* values are reported. **a-g.**

Results are representative of two independent *in vivo* studies. **h.** Representative image of the flow cytometry gating strategy used to assess phosphorylation of MYPT1 in PBMCs for Figures 6b and 8b using p-MYPT1-A647 antibody. **i.** Representative image of the flow cytometry gating strategy used to assess phosphorylation of MYPT1 in BMMCs for Figures 6f-g and S8c using CD71-PE/Cy7 and p-MYPT1-A647 antibodies. Source data are provided as a Source Data file.

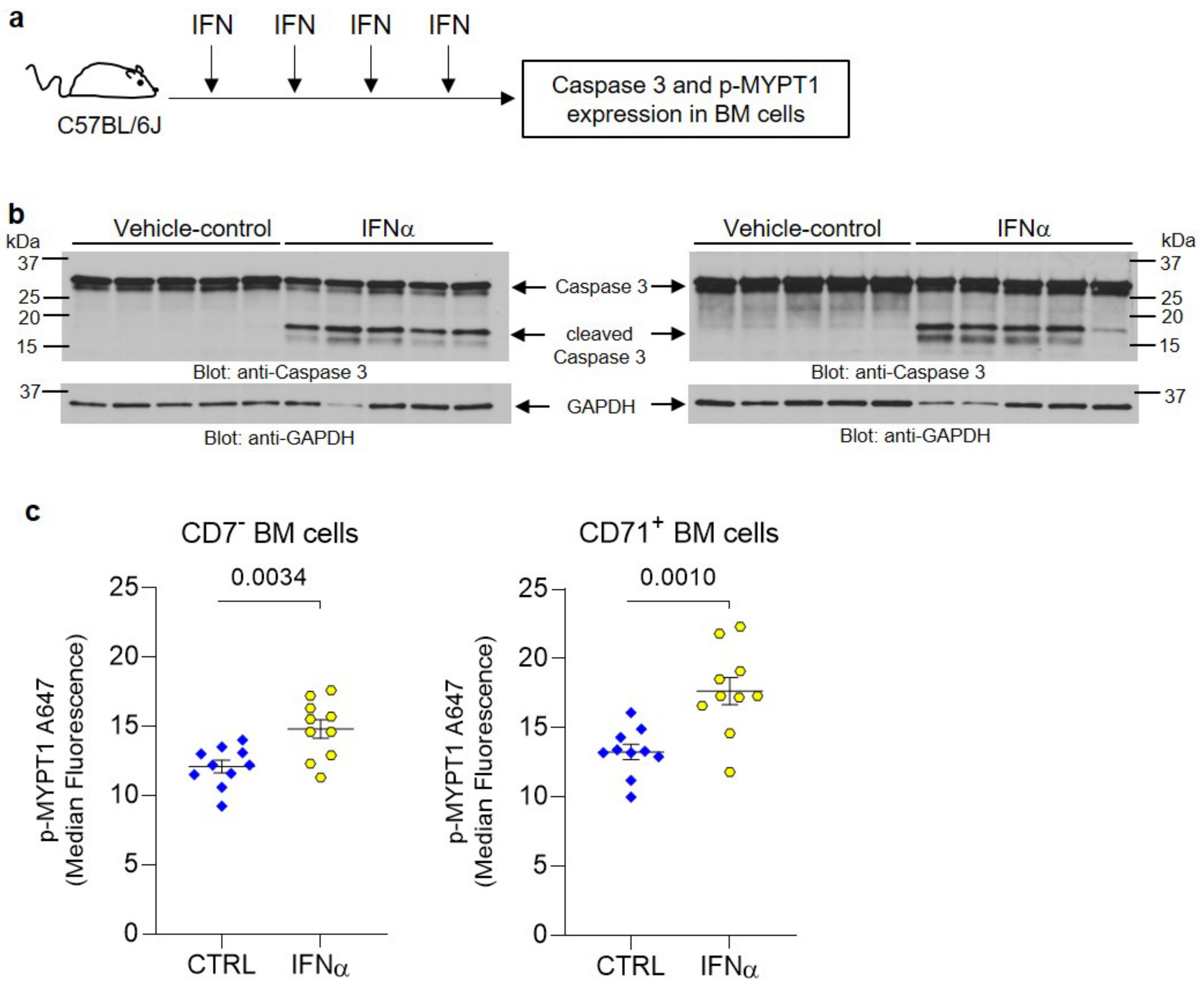

Supplementary Fig. 8. IFNα treatment induces cleavage of caspase 3 and phosphorylation of the ROCK downstream target MYPT1 *in vivo*. Related to Fig. 6.

a-c. C57BL/6J wild-type mice were treated with murine PEG-IFNα once a week (600 ng/mouse, subcutaneous injection) for four weeks or vehicle (PBS). **a.** Schematic illustration of the *in vivo* mouse model used to test the effects of PEG-IFNα treatment on caspase 3 activation and MYPT1 phosphorylation in bone marrow (BM) cells. **b.** Immunoblotting analysis of caspase 3 in lysates from bone marrow cells isolated from vehicle-control (n = 10) and IFNα-treated (n = 10) C57BL/6J mice, as indicated. Source data are provided as a Source Data file. **c.** Scatter dot plots show median fluorescence of phosphorylated MYPT1 (p-MYPT1 A647) in CD71 negative (CD71-) and

CD71 positive (CD71+) bone marrow mononuclear cells collected twenty-four hours after the fourth dose of PEG-IFNα (IFNα n = 10, yellow) or PBS (CTRL n = 10, blue). Data were assessed by flow cytometry and the gating strategy used is shown in Supplementary Figure 7i. Statistical analyses were performed using two-samples two-tailed t-test between CTRL and IFNα-treated groups. *p* values are reported. Source data are provided as a Source Data file.

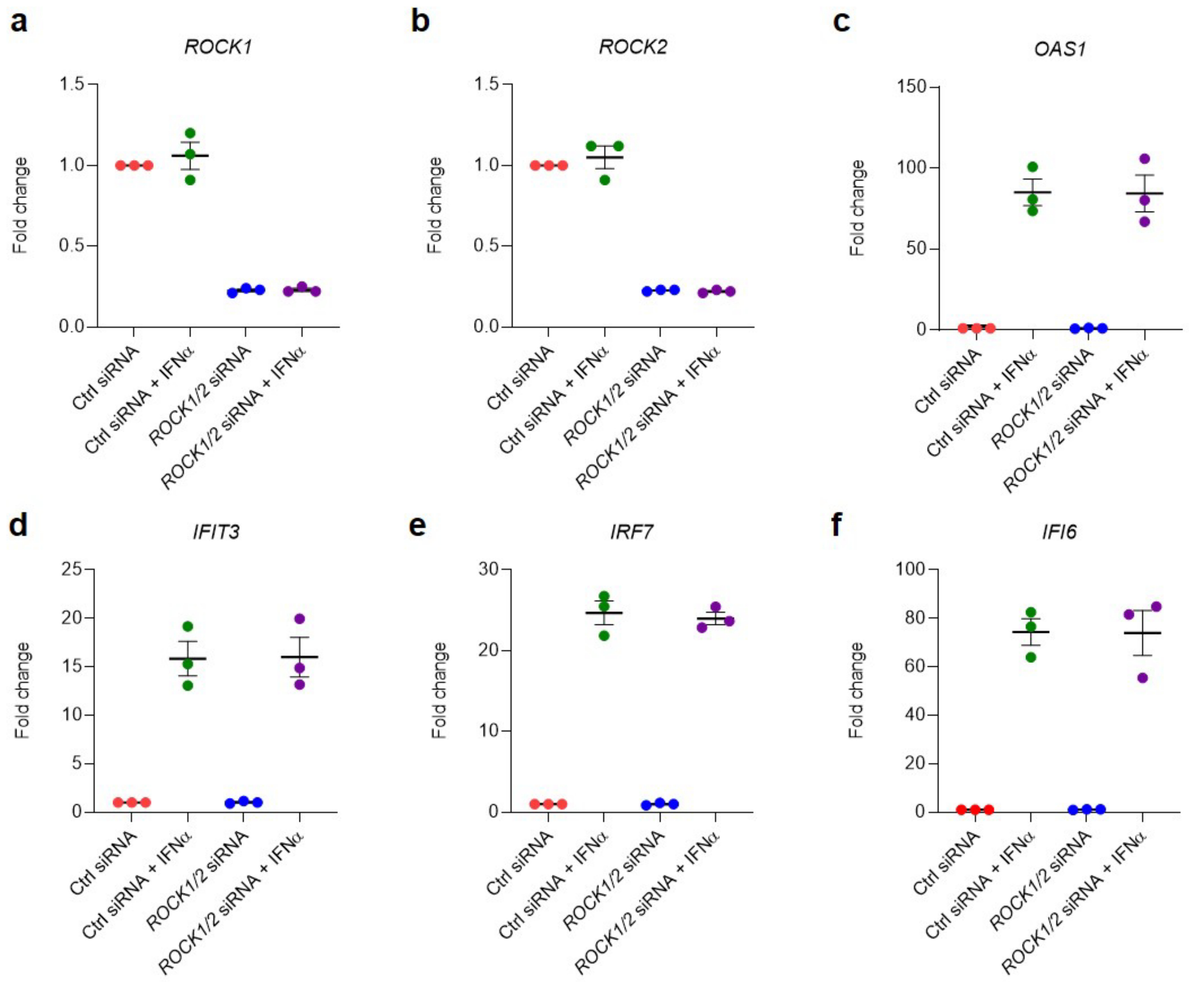

Supplementary Fig. 9. siRNA-mediated knockdown of *ROCK1/2* does not affect IFNα-induced expression of ISGs.

qRT-PCR analysis of (**a**) *ROCK1*, (**b**) *ROCK2*, (**c**) *OAS1*, (**d**) *IFIT3*, (**e**) *IRF7* and (**f**) *IFI6* mRNA expression in control (Ctrl) siRNA or ROCK1 and ROCK2 (*ROCK1/2*) siRNA transfected *JAK2*^{V617F}-positive HEL cells, either left untreated or treated with IFNα (1000 IU/mL) for 24 hours. GAPDH expression was used for normalization and to calculate delta Ct (ΔCt) for each gene for each experimental condition. The mRNA expression was calculated using the ΔΔCt method, and the data were plotted as the increase in fold change compared with Ctrl siRNA untreated samples for each independent experiment. Data are means ± SEM from three independent experiments. Source data are provided as a Source Data file.

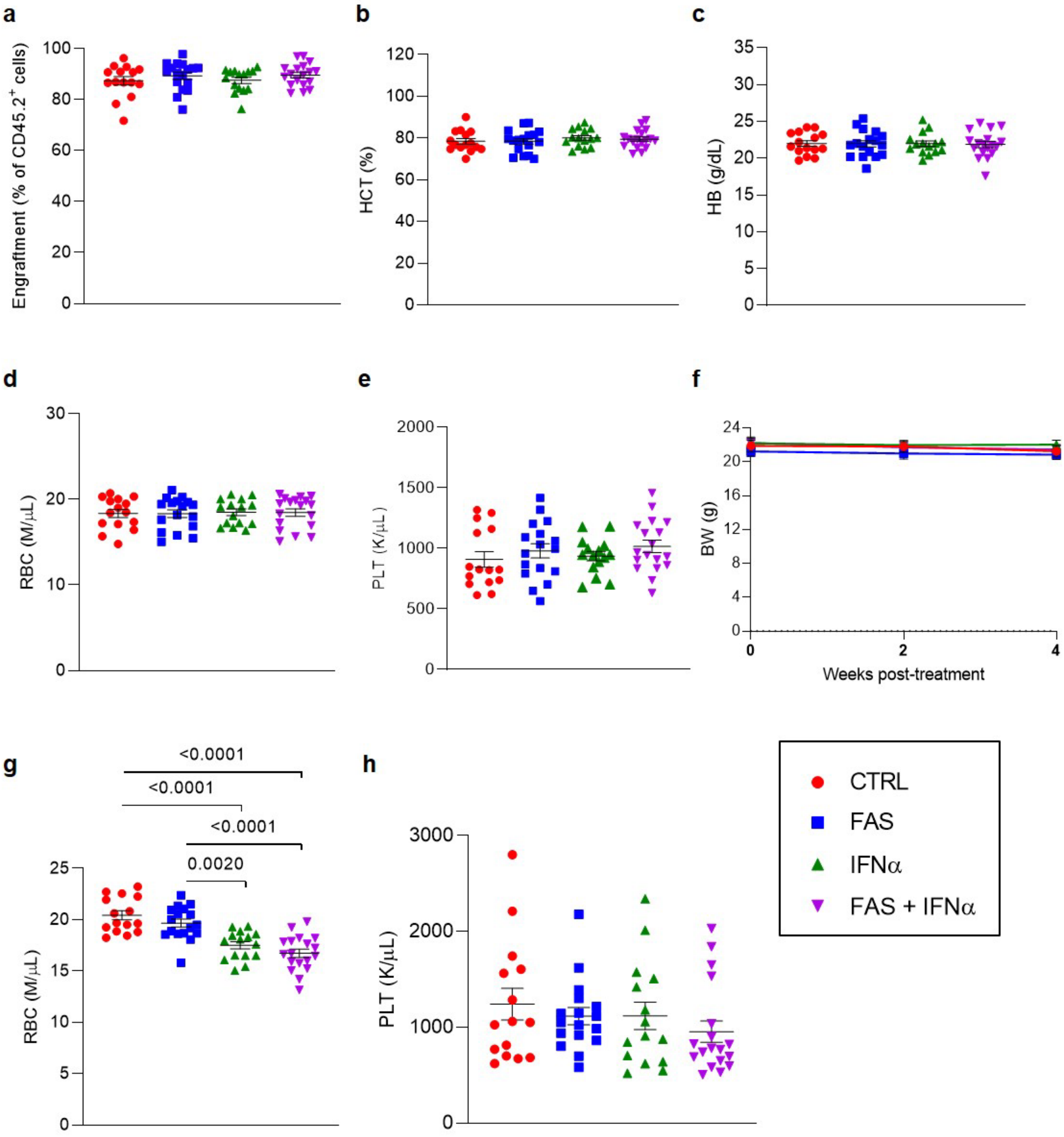

Supplementary Fig. 10. Engraftment, hematocrit, hemoglobin, red blood cells, platelet counts and body weight prior to and post PEG-IFNα and/or Fasudil treatment. Related to Fig. 8.

a-h. CD45.1 mice were transplanted with $Jak2^{V617F/+}$ KI bone marrow cells isolated from $Jak2^{V617F/+}$VavCre+ CD45.2 donor mice. Five weeks later, peripheral blood was collected and recipient mice were randomized by engraftment, hematocrit, and hemoglobin counts into four

treatment groups: Vehicles-control (CTRL), Fasudil (FAS), PEG-IFNα (IFNα) and Fasudil combined with PEG-IFNα (FAS + IFNα). CD45.1 recipient mice were treated with PEG-IFNα once a week, and/or Fasudil four times per week or vehicles (PBS) for four weeks. Data shown are pooled from four independent studies (CTRL n = 15, FAS n = 17, IFNα n = 15, FAS + IFNα n = 18); each independent study performed using 3-6 mice per treatment group. **a-e.** Scatter dot plots show (**a**) engraftment (% of PerCP-Cy5.5 $CD45.2^{+}$ cells in peripheral blood, the gating strategy used is shown in Figure S6a), (**b**) hematocrit (HCT), (**c**) hemoglobin (HB), (**d**) red blood cells (RBC) and (**e**) platelet (PLT) counts of recipients from each treatment group before initiation of treatments. **f.** Mouse body weight (BW) was recorded before and after initiation of treatment, as indicated. **g-h.** Scatter dot plots show (**g**) RBC and (**h**) PLT counts of recipients from each treatment group after four weeks of treatment. **a-h.** Data shown are means ± SEM. Statistical analyses were performed using one-way ANOVA followed by Tukey's multiple comparisons test. Statistical significant *p* values are reported. Source data are provided as a Source Data file.

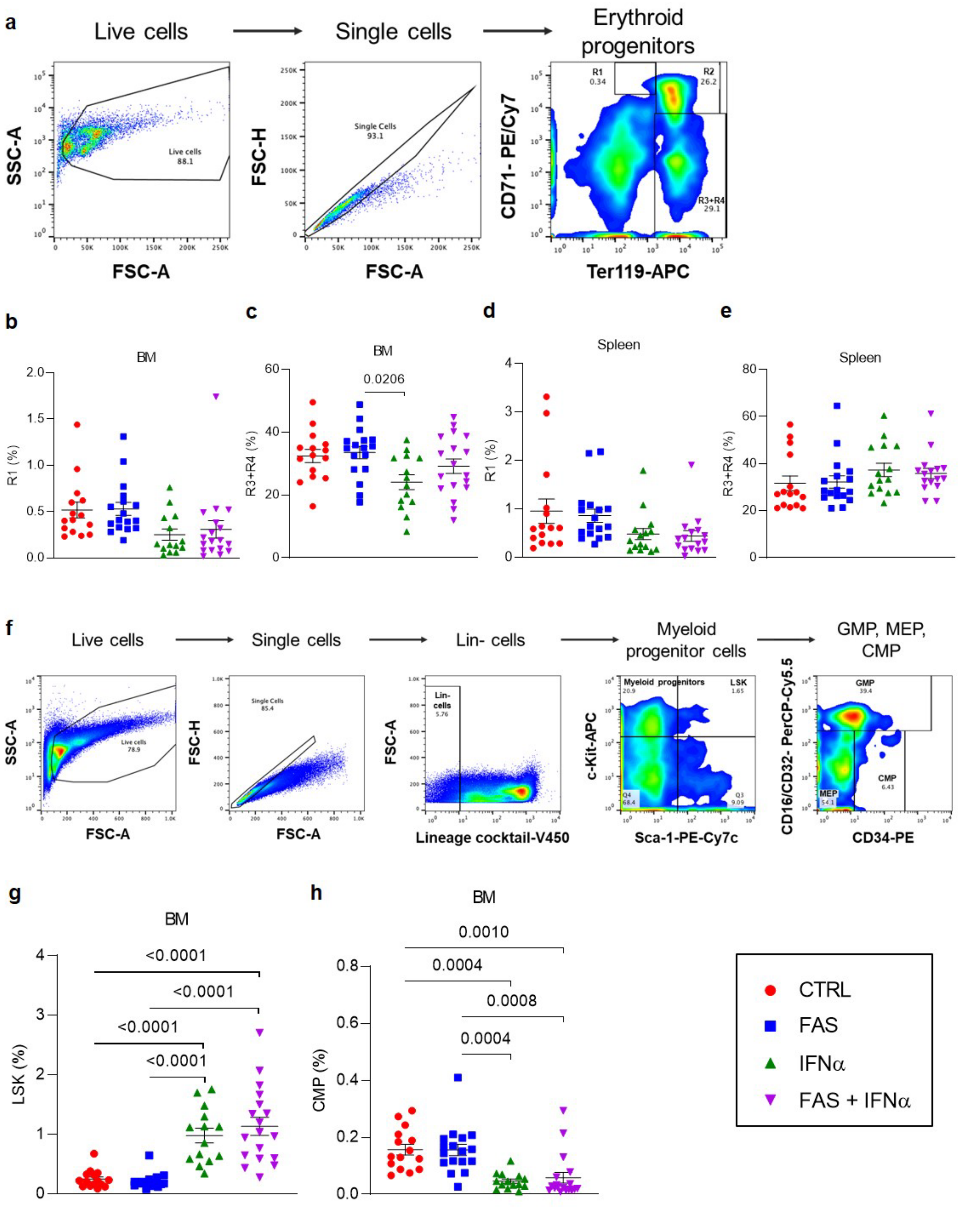

Supplementary Fig. 11. Effects of Fasudil and IFNα combination treatment on hematopoietic progenitor cells. Related to Fig. 8.

a-h. Effects of Fasudil (FAS) and/or PEG-IFNα (IFNα) treatment on bone marrow (BM) and spleen hematopoietic progenitor cells in *Jak2*$^{V671F/+}$ KI mouse model. Data shown are pooled from four independent studies (for BM samples: CTRL, n = 15; FAS, n = 17; IFNα, n = 14; FAS + IFNα, n = 18; for spleen samples CTRL, n = 15; FAS, n = 17; IFNα, n = 15; FAS + IFNα, n = 16). Each independent study was performed using 3-6 mice per treatment group. Data shown are means ± SEM assessed by flow cytometry analyses. **a.** Representative image of the flow cytometry gating strategy used to assess treatment effects on abundance of erythroid progenitors for Figures 8f-g and S11b-e using Ter119 and CD71 antibodies. R1, Ter119^{med}CD71^{high} (proerythroblasts); R2, Ter119^{high}CD71^{high} (basophilic erythroblasts); R3, Ter119^{high}CD71^{med} (late basophilic and polychromatophilic erythroblasts) and R4, Ter119^{high}CD71^{low} (orthochromatophilic erythroblasts)[2]. **b-e.** Percentage of R1 and R3+R4 erythroblasts in BM and spleen. **f.** Representative image of the flow cytometry gating strategy used to assess treatment effects on abundance of myeloid progenitor cells for Figures 8h-j and S11g-h using Lineage cocktail, c-Kit, Sca-1, CD34 and CD16/CD32 antibodies. Lin-, Lineage negative cells; LSK, Lin^{-} Sca-1^{+} c-Kit^{+} cells; GMP, granulocyte-monocyte progenitors; MEP, megakaryocyte-erythroid progenitors and CMP, common myeloid progenitors. **g-h.** Percentage of BM (**g**) LSKs and (**h**) CMPs. Statistical analyses were performed using one-way ANOVA followed by Tukey's multiple comparisons test. Statistical significant *p* values are reported. Source data are provided as a Source Data file.

Supplementary References

1. Perez-Riverol, Y. et al. The PRIDE database and related tools and resources in 2019: improving support for quantification data. *Nucleic Acids Res*. **47**, D442-D450 (2019).
2. Socolovsky, M., Nam, H., Fleming, M. D., Haase, V. H., Brugnara, C. & Lodish, H. F. Ineffective erythropoiesis in Stat5a(-/-)5b(-/-) mice due to decreased survival of early erythroblasts. *Blood* **98**, 3261-3273 (2001).
