# Supplementary material for: Discovery of a signaling feedback circuit that defines interferon responses in myeloproliferative neoplasms
Source: Nat Commun. 2022 Apr 1;13:1750. doi: 10.1038/s41467-022-29381-7 (PMC8975834; doi:10.1038/s41467-022-29381-7)
Supplement: Supplementary file 2 — Reporting summary [file 41467_2022_29381_MOESM2_ESM.pdf]

nature portfolio

Corresponding author(s): Leonidas C. Platanias

Last updated by author(s): January 31, 2022

Reporting Summary

Nature Portfolio wishes to improve the reproducibility of the work that we publish. This form provides structure for consistency and transparency in reporting. For further information on Nature Portfolio policies, see our Editorial Policies and the Editorial Policy Checklist.

Statistics

For all statistical analyses, confirm that the following items are present in the figure legend, table legend, main text, or Methods section.

n/a	Confirmed	
☐	☒	The exact sample size (*n*) for each experimental group/condition, given as a discrete number and unit of measurement
☐	☒	A statement on whether measurements were taken from distinct samples or whether the same sample was measured repeatedly
☐	☒	The statistical test(s) used AND whether they are one- or two-sided<br>*Only common tests should be described solely by name; describe more complex techniques in the Methods section.*
☐	☒	A description of all covariates tested
☐	☒	A description of any assumptions or corrections, such as tests of normality and adjustment for multiple comparisons
☐	☒	A full description of the statistical parameters including central tendency (e.g. means) or other basic estimates (e.g. regression coefficient) AND variation (e.g. standard deviation) or associated estimates of uncertainty (e.g. confidence intervals)
☐	☒	For null hypothesis testing, the test statistic (e.g. *F*, *t*, *r*) with confidence intervals, effect sizes, degrees of freedom and *P* value noted<br>*Give P values as exact values whenever suitable.*
☒	☐	For Bayesian analysis, information on the choice of priors and Markov chain Monte Carlo settings
☒	☐	For hierarchical and complex designs, identification of the appropriate level for tests and full reporting of outcomes
☒	☐	Estimates of effect sizes (e.g. Cohen's *d*, Pearson's *r*), indicating how they were calculated

Our web collection on statistics for biologists contains articles on many of the points above.

Software and code

Policy information about availability of computer code

Data collection	Gene Expression Omnibus (GEO), Bio-Rad CFX Maestro software, BD FACSDiva, Hemavet 950 software, Mascot 2.5 (Matrix Science), Gen5 Software (Microplate reader software), DepMap portal (https://depmap.org/portal/), human SwissProt database (https://www.uniprot.org/uniprot/?query=reviewed:yes)
Data analysis	GraphPad Prism 8, FlowJo v10.6, R package lme4 version 1.1.26, Scaffold 4 proteome software, BLAST - blastn, ImageJ

For manuscripts utilizing custom algorithms or software that are central to the research but not yet described in published literature, software must be made available to editors and reviewers. We strongly encourage code deposition in a community repository (e.g. GitHub). See the Nature Portfolio guidelines for submitting code & software for further information.

Data

Policy information about availability of data

All manuscripts must include a data availability statement. This statement should provide the following information, where applicable:

- Accession codes, unique identifiers, or web links for publicly available datasets
- A description of any restrictions on data availability
- For clinical datasets or third party data, please ensure that the statement adheres to our policy

The mass spectrometry proteomics data generated in this study have been deposited to the ProteomeXchange Consortium via the PRIDE (76) partner repository with the dataset identifier PXD021748 and 10.6019/PXD021748 (http://proteomecentral.proteomexchange.org/cgi/GetDataset?ID=PXD021748). The ROCK1/2 gene expression data in MPN patients versus healthy individuals used in this study are available in the NCBI GEO database under accession code GSE54646 (https://www.ncbi.nlm.nih.gov/geo/query/acc.cgi?acc=GSE54646) and within the Source Data file of this manuscript. The proteomic data for ROCK1 (Q13464) (https://depmap.org/portal/gene/ROCK1?tab=characterization&characterization=proteomics) and ROCK2 (O75116) (https://depmap.org/portal/gene/ROCK2?

tab=characterization&characterization=proteomics) proteins are available in the public DepMap portal and within the Source Data file of this manuscript. The authors declare that all other data that support the findings of this study are available within the paper, its supplementary information, or Source Data file.

Field-specific reporting

Please select the one below that is the best fit for your research. If you are not sure, read the appropriate sections before making your selection.

- [x] Life sciences
- [ ] Behavioural & social sciences
- [ ] Ecological, evolutionary & environmental sciences

For a reference copy of the document with all sections, see nature.com/documents/nr-reporting-summary-flat.pdf

Life sciences study design

All studies must disclose on these points even when the disclosure is negative.

Sample size	Group sample sizes of 5 and 5 achieve 80% power to reject the null hypothesis of equal means when the population mean difference is 60.7 with a standard deviation for both groups of 30.0 and with a significance level (alpha) of 0.05 using a two-sided two-sample equal-variance t-test. For the in vivo studies shown in Figure 5a, 6a and S8a we used at least 5 animals per treatment group. For the in vivo studies shown in Figure 8a, we were limited to using 3-6 mice per treatment group as the number of recipient mice that we are able to transplant is dependent on the number of the same sex and age of Jak2VF/+VavCre+ donor mice that we can generate by breeding. To increase the statistical power of our study, we combined the data from four independent studies in the same plot for the different parameters studied.<br>No sample size calculation was performed to predetermine sample size for the in vitro experiments. For experiments using cell lines, we have used three or more independent biological replicates for each experimental condition to assess differences between 2 or more experimental groups. For experiments using MPN-patient derived primary cells, we have used cells isolated from at least three different patients for each experimental condition to assess differences between 2 or more experimental groups. The sample sizes used were sufficient to demonstrate large differences between experimental conditions.
Data exclusions	For the in vivo studies, mice were excluded from analysis if they were sacrificed or died for reasons unrelated to MPN-phenotype or treatment (e.g. few mice were excluded due to signs of sickness not related to MPN-phenotype or treatment). For the flow cytometry results shown in Figure 8 and S11 there were not enough bone marrow cells to collect data from one mouse in the IFN-treated group and there were not enough spleen hematopoietic cells to collect data from two mice from the IFN+Fasudil treatment group.
Replication	The in vitro experiments were repeated independently at least three times, except for those specifically indicated in the figure legends. Experiments involving statistical analyses were performed using at least three independent biological replicates, or using samples from at least three different patients/individuals or from at least three different mice for each experimental condition. The animal studies involving transplant models were repeated at least two independent times and the study shown in Figure S8 was performed once using 10 mice per treatment group. For all experiments, all attempts at replication were successful.
Randomization	Animals were randomized into treatment groups by hematocrit and/or hemoglobin levels upon confirmation of engraftment (for the transplant models) and Polycythemia vera-like phenotype (for the Jak2VF KI mouse models). For the animal study shown in Figure S8, animals were randomized into treatment groups by body weight. No particular randomization method was used for other experiments. The same number of cells were plated for each treatment condition and same amount of protein was used for co-IP and immunoblotting analyses for each experimental condition. Primary MPN patient samples were randomly selected.
Blinding	For the in vivo studies, collection of data was blinded, but to calculate differences between treatment groups each animal number had to be matched to its treatment. For the qPCR analyses using patient samples, the investigators were blinded to group allocation during data collection, the type of response was matched to each sample number during data analysis. All other experiments were performed in a non-blinded manner, because the experimental design was complicated and blinding feasibility was poor.

Reporting for specific materials, systems and methods

We require information from authors about some types of materials, experimental systems and methods used in many studies. Here, indicate whether each material, system or method listed is relevant to your study. If you are not sure if a list item applies to your research, read the appropriate section before selecting a response.

Materials & experimental systems

n/a	Involved in the study	
[ ]	[x]	Antibodies
[ ]	[x]	Eukaryotic cell lines
[x]	[ ]	Palaeontology and archaeology
[ ]	[x]	Animals and other organisms
[ ]	[x]	Human research participants
[ ]	[x]	Clinical data
[x]	[ ]	Dual use research of concern

Methods

n/a	Involved in the study	
[x]	[ ]	ChIP-seq
[ ]	[x]	Flow cytometry
[x]	[ ]	MRI-based neuroimaging

Antibodies

Antibodies used	From Cell Signaling Technology: Rabbit monoclonal anti-ULK1 (clone D8H5) Cat # 8054 (WB 1:1000, co-IP 2.1µg/mg protein), Rabbit monoclonal anti-PKC-delta (clone D10E2) Cat # 9616 (WB 1:1000, IP: 0.6µg/mg protein), Rabbit monoclonal antibody IgG XP Isotype Control (clone DA1E) Cat # 3900 (control for co-IP ULK1 2.1µg/mg protein, control for IP PKC-delta 0.6µg/mg protein), Rabbit polyclonal anti-GST Cat # 2622 (WB 1:1000), Rabbit monoclonal anti-Phospho-p38 MAPK (Thr180/Tyr182) (clone 3D7) Cat # 9215 (WB 1:1000), Rabbit monoclonal anti-p38 MAPK (clone D13E1) XP Cat # 8690 (WB 1:1000), Rabbit monoclonal anti-ROCK1 (clone C8F7) Cat # 4035 (WB 1:1000), Rabbit monoclonal anti-ROCK2 (clone D1B1) Cat # 9029 (WB 1:1000), rabbit monoclonal anti-phospho-ULK1 (Ser757) (clone D7O6U) Cat # 14202 (WB 1:1000), rabbit polyclonal anti-caspase 3 Cat # 9662 (WB 1:1000), and rabbit polyclonal anti-PARP Cat # 9542 (WB 1:1000).<br>From Proteintech: rabbit polyclonal anti-PKC Delta Cat # 14188-1-AP (WB 1:1000).<br>From EMD Millipore: Mouse monoclonal anti-GAPDH clone 6C5, Cat # MAB374 (WB 1:20,000).<br>From Santa Cruz Biotechnology: Mouse monoclonal anti- p-MYPT1 (clone F-11) Alexa Fluor 647 Cat # sc-377531 AF647 (Flow cytometry [F] 1:50), Normal mouse IgG1 Alexa Fluor 647 Cat # sc-24636 (F 1:50), c-Myc antibody (clone 9E10) conjugated to agarose beads Cat # sc-40 AC (IP 500 µg/ml).<br>From BD Biosciences: PerCP-Cy5.5 mouse anti-mouse CD45.2 (Clone 104; BD Pharmigen) Cat # 561096 (F 1:200), V450 mouse anti-mouse CD45.1 (Clone A20; BD Horizon) Cat # 560520 (F 1:200), V450 Mouse Lineage Antibody Cocktail Cat # 561301 (Components include clone 500A2, which recognizes Mouse CD3e; M1/70, which recognizes CD11b; RA3-6B2, which recognizes CD45R/B220; TER-119, which recognizes Ly-76, mouse erythroid cells; and RB6-8C5, which recognizes Ly-6G and Ly-6C) (F 1:20), APC Rat Anti-Mouse CD117 (c-kit) (Clone 2B8) antibody Cat # 553356 (F 1:100), PE Rat anti-Mouse CD34 (Clone RAM34) antibody Cat # 551387 (F 1:40), and PerCP-Cy5.5 Rat Anti-Mouse CD16/CD32 (Clone 2.4G2) antibody Cat # 560540 (F 1:200).<br>From Biolegend: PE/Cyanine7 anti-mouse CD71 (clone RI7217) antibody Cat # 113812 (F 1:200), APC anti-mouse TER-119/Erythroid Cells (clone TER-119) antibody Cat # 116212 (F 1:200).<br>From Invitrogen/Thermo Fisher Scientific: Ly-6A/E (Sca-1) Monoclonal Antibody (clone D7), PE-Cyanine7, eBioscience Cat # 25-5981-82 (F 1:100).<br>Custom-made antibodies by Pierce Biotechnology/ Thermo Fisher Scientific: Rabbit polyclonal anti-Phospho-ULK1 (Ser 341) (WB 1:1000) and Rabbit polyclonal anti-Phospho ULK1 (Ser 495) (WB 1:500).
Validation	Search RRID antibody numbers (where available) in the RRID Portal (https://scicrunch.org/resources), the Resource Identification Portal, supporting guidelines for Rigor and Transparency in scientific publications, to get more detailed information on antibodies used. Manufacturers' web-page link for each antibody is also provided with validation information.<br>Rabbit monoclonal anti-ULK1 = RRID AB_11178668, https://www.cellsignal.com/products/primary-antibodies/ulk1-d8h5-rabbit-mab/8054<br>Rabbit monoclonal anti-PKC-delta = RRID AB_10949973, https://www.cellsignal.com/products/primary-antibodies/pkcd-d10e2-rabbit-mab/9616<br>Rabbit monoclonal antibody IgG XP Isotype Control = RRID AB_1550038, https://www.cellsignal.com/products/primary-antibodies/rabbit-da1e-mab-igg-xp-isotype-control/3900<br>Rabbit monoclonal anti-Phospho-p38 MAPK = RRID AB_331762, https://www.cellsignal.com/products/primary-antibodies/phospho-p38-mapk-thr180-tyr182-3d7-rabbit-mab/9215<br>Rabbit monoclonal anti-p38 MAPK = RRID AB_10999090, https://www.cellsignal.com/products/primary-antibodies/p38-mapk-d13e1-xp-rabbit-mab/8690<br>Rabbit polyclonal anti-GST = RRID AB_331670, https://www.cellsignal.com/products/primary-antibodies/gst-tag-antibody/2622<br>Rabbit monoclonal anti-ROCK1 = RRID AB_2238679, https://www.cellsignal.com/products/primary-antibodies/rock1-c8f7-rabbit-mab/4035<br>Rabbit monoclonal anti-ROCK2 = RRID AB_11127802, https://www.cellsignal.com/products/primary-antibodies/rock2-d1b1-rabbit-mab/9029?site-search-type=Products&N=4294956287&Ntt=rock2&fromPage=plp<br>Rabbit monoclonal anti-phospho-ULK1 (Ser757) (D7O6U) = https://www.cellsignal.com/products/primary-antibodies/phospho-ulk1-ser757-d7o6u-rabbit-mab/14202<br>Rabbit polyclonal anti-caspase 3 = https://www.cellsignal.com/products/primary-antibodies/caspase-3-antibody/9662<br>Rabbit polyclonal anti-PARP = RRID:AB_2160739, https://www.cellsignal.com/products/primary-antibodies/parp-antibody/9542<br>Rabbit polyclonal anti-PKC Delta = RRID:AB_10638614, https://www.ptglab.com/products/PRKCD-Antibody-14188-1-AP.htm<br>Mouse monoclonal anti-GAPDH = RRID:AB_2107445, https://www.emdmillipore.com/US/en/product/Anti-Glyceraldehyde-3-Phosphate-Dehydrogenase-Antibody-clone-6C5,MM_NF-MAB374<br>Mouse monoclonal anti- p-MYPT1 (F-11) Alexa Fluor 647 = https://www.scbt.com/p/p-mypt1-antibody-f-11<br>Normal mouse IgG1 Alexa Fluor 647 = RRID:AB_737215, https://www.scbt.com/p/normal-mouse-igg1-alexa-fluor-647<br>c-Myc antibody (9E10) conjugated to agarose beads = https://www.scbt.com/p/c-myc-antibody-9e10<br>PerCP-Cy5.5 mouse anti-mouse CD45.2 = RRID:AB_2034008, https://www.bdbiosciences.com/en-nz/products/reagents/flow-cytometry-reagents/research-reagents/single-color-antibodies-ruo/percp-cy-5-5-mouse-anti-mouse-cd45-2.561096<br>V450 mouse anti-mouse CD45.1 = RRID:AB_1727490, https://www.bdbiosciences.com/en-nz/products/reagents/flow-cytometry-reagents/research-reagents/single-color-antibodies-ruo/v450-mouse-anti-mouse-cd45-1.560520<br>V450 Mouse Lineage Antibody Cocktail = https://www.bdbiosciences.com/en-us/products/reagents/flow-cytometry-reagents/research-reagents/panels-multicolor-cocktails-ruo/v450-mouse-lineage-antibody-cocktail-with-isotype-control.561301<br>APC Rat Anti-Mouse CD117 (c-kit) = https://www.bdbiosciences.com/en-us/products/reagents/flow-cytometry-reagents/research-reagents/single-color-antibodies-ruo/apc-rat-anti-mouse-cd117.553356<br>PE Rat anti-Mouse CD34 = https://www.bdbiosciences.com/en-us/products/reagents/flow-cytometry-reagents/research-reagents/single-color-antibodies-ruo/pe-rat-anti-mouse-cd34.551387<br>PerCP-Cy5.5 Rat Anti-Mouse CD16/CD32 = RRID:AB_1645259, https://www.bdbiosciences.com/en-nz/products/reagents/flow-cytometry-reagents/research-reagents/single-color-antibodies-ruo/percp-cy-5-5-rat-anti-mouse-cd16-cd32.560540<br>PE/Cyanine7 anti-mouse CD71 = RRID:AB_2203382, https://www.biolegend.com/en-us/products/pe-cyanine7-anti-mouse-cd71-antibody-6185?Clone=RI7217<br>APC anti-mouse TER-119/Erythroid Cells = RRID:AB_313713, https://www.biolegend.com/en-us/products/apc-anti-mouse-ter-119-

erythroid-cells-antibody-1863?Clone=TER-119
Ly-6A/E (Sca-1) Monoclonal Antibody (D7), PE-Cyanine7 = RRID:AB_469669, https://www.thermofisher.com/antibody/product/Ly-6A-E-Sca-1-Antibody-clone-D7-Monoclonal/25-5981-82
The Rabbit polyclonal anti-Phospho-ULK1 (Ser 341) and Rabbit polyclonal anti-Phospho ULK1 (Ser 495) antibodies were generated and validated as described in the Methods section. The results of the validation are presented in Supplementary Figure 1.

Eukaryotic cell lines

Policy information about cell lines

Cell line source(s)	HEL (TIB-180; ATCC), KT-1 (RRID:CVCL_D200) from Dr. Fujita's lab (ref. 65), U937 (CRL-1593.2; ATCC), SET-2 cells (ACC-608; DSMZ), HEK293T cells (CRL-3216; ATCC), immortalized Ulk1/2+/+ and Ulk1/2-/- MEFs (ref. 66) from Dr. Thompson's lab.
Authentication	Human cell lines, available through ATCC and DSMZ, were tested by STR analyses and matched to the respective database. The expression status of Ulk1 and Ulk2 in the Ulk1/2+/+ (wild-type) and Ulk1/2-/- (double knockout) MEFs was confirmed by immunoblotting and/or qPCR analyses. KT-1 cells have not been authenticated for lack of a published STR profile, to the best of our knowledge. We monitor this cell line based on cell morphology and interferon-responsiveness.
Mycoplasma contamination	All cell lines tested negative for mycoplasma contamination.
Commonly misidentified lines (See ICLAC register)	Misidentified cell lines were not used.

Animals and other organisms

Policy information about studies involving animals; ARRIVE guidelines recommended for reporting animal research

Laboratory animals	10-11 week old female and male CD45.1 (B6.SJL-PtprcaPepcb/BoyJ, Stock No. 002014, Jackson Laboratory) mice (C57BL/6) were used as recipients and 6-10 week old female and male Jak2+/+VavCre- and Jak2V617F/+ VavCre+ donor mice (C57BL/6) were used as donors for the transplant studies. Both female and male (6-12 weeks old) Jak2V617F conditional knock-in mice (CD45.2) (ref. 30, provided by B. L. Ebert and A. Mullally - Harvard Medical School) and Vav-Cre+ (CD45.2) (B6.Cg-Commd10Tg(Vav1-icre)A2Kio/J, Stock No: 008610, Jackson Laboratory) mice were used for breeding to generate Jak2+/+VavCre- and Jak2V617F/+ Vav-cre+ donor mice. 9-10 week old female C57BL/6J mice (The Jackson Laboratory, Stock No: 000664) were used for the studies to assess the effects of IFN treatment in wild-type mice in vivo.
Wild animals	The study did not involve wild animals.
Field-collected samples	The study did not involve samples collected from the field.
Ethics oversight	All animal studies were approved by the Northwestern University Institutional Animal Care and Use Committee (Protocol # IS00012751) and conducted in accordance with the Guidelines for the Care and Use of Laboratory Animals.

Note that full information on the approval of the study protocol must also be provided in the manuscript.

Human research participants

Policy information about studies involving human research participants

Population characteristics	For the study correlating expression of genes of interest with clinical outcomes to interferon treatment, primary samples collected before treatment from 18 patients were randomly selected with the assistance of the MPN-RC Biostatistics Core C and provided by the MPN-RC Tissue Bank Core B for our analysis. MPN patients had been enrolled in the MPD-RC-111 study, which included patients with polycythemia vera and essential thrombocythemia that were refractory or intolerant to hydroxyurea therapy (clinical trial #NCT01259817) after providing informed consent. 15 patients were diagnosed as having polycythemia vera (PV) while 3 patients had essential thrombocythemia (9 men and 9 women, between the ages of 36 and 84 years). The clinical characteristics of each patient are provided in Supplementary Table 1. Responses were assessed after 12 and 24 months of treatment according to criteria established by the European LeukemiaNET. There were 9 responders and 9 non-responders analyzed. A more detailed description of participants enrolled in the clinical trial #NCT01259817 and criteria for assessment of clinical response have been previously described (Yacoub A., et al. Blood (2019) 134 (18): 1498–1509).
Recruitment	The criteria for enrollment of patients in clinical trial #NCT01259817 have been previously described (Yacoub A., et al. Blood (2019) 134 (18): 1498–1509). Samples for banking and for the in vitro colony studies were obtained from MPN patients seen for evaluation, follow-up, and/or treatment in the clinics of the respective institutions, after informed consent was obtained. We then received de-identified samples, selected randomly and unbiased, from the tumor banks. Participants did not receive any compensation.
Ethics oversight	Collection of samples after obtaining informed consent was approved by the respective Institutional Review Boards of Institutions participating in the MPN Research Consortium or the IRB of Northwestern University, in accordance with the Declaration of Helsinki protocol. For the clinical trial #NCT01259817 samples, studies were approved as described in Yacoub A., et al. Blood (2019) 134 (18): 1498–1509.

Note that full information on the approval of the study protocol must also be provided in the manuscript.

Clinical data

Policy information about clinical studies

All manuscripts should comply with the ICMJE guidelines for publication of clinical research and a completed CONSORT checklist must be included with all submissions.

Clinical trial registration	NCT01259817
Study protocol	Yacoub, A. et al. Pegylated interferon alfa-2a for polycythemia vera or essential thrombocythemia resistant or intolerant to hydroxyurea. Blood 134, 1498-1509 (2019)
Data collection	In our study we used peripheral blood or bone marrow-mononuclear frozen samples banked at the New York Blood Center and that had been collected prior to treatment initiation from patients enrolled in the Myeloproliferative Disorders Research Consortium (MPD-RC)-111 study who received PEG-IFN-alpha2a (Pegasys) (clinical trial #NCT01259817). We measured the mRNA levels of PKC-delta, ULK1 and p38 MAPK in these samples by qRT-PCR (Fig. 3b). 18S mRNA levels were used as reference/normalization control.
Outcomes	After data analyses, we matched the mRNA levels of PKC-delta, ULK1 and p38 MAPK with the response to IFN treatment of each MPN patient (Yacoub, A. et al. Pegylated interferon alfa-2a for polycythemia vera or essential thrombocythemia resistant or intolerant to hydroxyurea. Blood 134, 1498-1509 (2019)). Patients expressing higher pre-treatment mRNA levels of ULK1 and p38 MAPK were more likely to respond to PEG-IFN-alpha2a therapy (Fig. 3b), from which we infer a key role for this pathway in the anti-neoplastic effects of IFN in vivo. Future prospective clinical studies will be required to fully validate these findings and examine the potential of ULK1 and p38 MAPK as biomarkers of IFN-responsiveness.

Flow Cytometry

Plots

Confirm that:

- [x] The axis labels state the marker and fluorochrome used (e.g. CD4-FITC).
- [x] The axis scales are clearly visible. Include numbers along axes only for bottom left plot of group (a 'group' is an analysis of identical markers).
- [x] All plots are contour plots with outliers or pseudocolor plots.
- [x] A numerical value for number of cells or percentage (with statistics) is provided.

Methodology

Sample preparation	To determine the efficiency of engraftment of CD45.2+ bone marrow cells, mice peripheral blood (PB) was collected in EDTA-coated tubes, red blood cells were lysed using 1X RBC lysis buffer (eBioscience 10X RBC Lysis Buffer, Thermo Fisher Scientific/ invitrogen, Cat # 00-4300-54), then cells were stained in FACS buffer (PBS, 0.5% BSA, 1 mM EDTA). To assess the effects of PEG-IFN and/or Fasudil treatments on phosphorylation of MYPT1 in PB mononuclear cells (PBMCs), PB was collected from mice in EDTA-coated tubes and red blood cells were lysed using 1X RBC lysis buffer. PBMCs were then fixed, permeabilized and stained using the BD Cytofix/Cytoperm Fixation/Permeabilization Solution Kit (# BD 554714), according to the manufacturer's instructions. To assess the effects of PEG-IFN treatment on phosphorylation of MYPT1 in BM mononuclear cells (BMMCs), BM cells were flushed, homogenized through a 70µm cell strainer, and red blood cells were lysed using 1X RBC lysis buffer. BMMCs were then fixed, permeabilized and stained using the BD Cytofix/Cytoperm Fixation/ Permeabilization Solution Kit (# BD 554714), according to the manufacturer's instructions. To assess the effects of PEG-IFN and/or Fasudil treatments on erythroid development, BM cells were flushed and homogenized through a 70µm cell strainer, and spleens were mechanically dissociated and homogenized through a 70µm cell strainer. BM and spleen cells were then stained in FACS buffer. To assess the effects of PEG-IFN and/or Fasudil treatments on myeloid progenitor cells, BM cells were flushed and homogenized through a 70µm cell strainer, red blood cells were lysed with 1X RBC lysis buffer and BM mononuclear cells were then stained in FACS buffer. Cells were stained using fluorochrome-conjugated antibodies as described in the "Flow cytometry analysis" methods section.
Instrument	LSRII flow cytometer by BD Biosciences
Software	Data were acquired using BD FACSDiva, and analyzed using FlowJo software V10 (Treestar, CA).
Cell population abundance	No sorting was performed.
Gating strategy	For all flow cytometry analysis, the live cells were gated based on the FSC-A and SSC-A; then out of the live cell gate, single cells were gated based on the FSC-H and FSC-A; next out of the single cells gate, the different analysis were performed:<br>1) Engrafted bone marrow cells were determined as CD45.1- and CD45.2+ cells (Fig. S6a).<br>2) Comparison of p-MYPT1 levels was done by overlay of p-MYPT1 histograms of single cells (Fig. S7h).<br>3) CD71+ and CD71- BMMCs were determined by positive versus negative staining using anti-CD71-PE/Cy7 antibody and then out of each cell population comparison of p-MYPT1 levels was done by overlay of p-MYPT1 histograms of single cells (Fig. S7i).<br>4) Erythroid progenitors were defined based on staining with anti-Ter119-APC and anti-CD71-PE/Cy7 antibodies as R1: Ter119medCD71high (proerythroblasts); R2, Ter119highCD71high (basophilic erythroblasts); R3, Ter119highCD71med (polychromatophilic erythroblasts) and R4, Ter119highCD71low (orthochromatophilic erythroblasts) (Fig. S11a).<br>5) To determine abundance of progenitor cells, lineage negative (Lin-) cells were gated based on negative staining using V450 Mouse Lineage Antibody Cocktail; next out of the Lin- cells gate, myeloid progenitors were gated as c-Kit+Sca- cells (using

anti-c-Kit-APC and anti-Sca-1-PE/Cy7c antibodies); then out of the myeloid progenitor cells gate, GMPs, MEPs and CMPs were determined as shown in Fig. S11f based on staining with anti-CD16/32-PerCP-Cy5.5 and anti-CD34-PE antibodies.
The border between positive and negative cell populations was defined using single color and unstained controls.

☒ Tick this box to confirm that a figure exemplifying the gating strategy is provided in the Supplementary Information.
